# Genomic Mining Reveals Deep Evolutionary Relationships between Bornaviruses and Bats

**DOI:** 10.3390/v7112906

**Published:** 2015-11-10

**Authors:** Jie Cui, Lin-Fa Wang

**Affiliations:** Program in Emerging Infectious Diseases, Duke-NUS Graduate Medical School, Singapore 169857, Singapore; linfa.wang@duke-nus.edu.sg

**Keywords:** genomic mining, endogenous bornaviruses, bats, virus-host interaction

## Abstract

Bats globally harbor viruses in order Mononegavirales, such as lyssaviruses and henipaviruses; however, little is known about their relationships with bornaviruses. Previous studies showed that viral fossils of bornaviral origin are embedded in the genomes of several mammalian species such as primates, indicative of an ancient origin of exogenous bornaviruses. In this study, we mined the available 10 bat genomes and recreated a clear evolutionary relationship of endogenous bornaviral elements and bats. Comparative genomics showed that endogenization of bornaviral elements frequently occurred in vesper bats, harboring EBLLs (endogenous bornavirus-like L elements) in their genomes. Molecular dating uncovered a continuous bornavirus-bat interaction spanning 70 million years. We conclude that better understanding of modern exogenous bornaviral circulation in bat populations is warranted.

## 1. Introduction

Exogenous bornavirus is a neurotropic and enveloped RNA virus, belonging to the family *Bornaviridae* of the order Mononegavirales [1]. Its nonsegmented negative-strand genome encodes six proteins: nucleoprotein (N), phosphoprotein (P), matrix protein (M), glycoprotein (G), RNA-dependent RNA polymerase (L), and accessory protein (X). Borna disease (BD) was first described as a fatal neurologic disease of horses and sheep in 1885 [2]. Borna disease virus (BDV) can gain access to the central nervous system (CNS) of hosts and causes persistent infection in warm-blooded animals [3]. Exogenous bornaviruses are known to infect mammals and birds such as a bornavirus, which was identified in psittacine birds with a fatal neurological disorder [4]. Recently, a snake bornavirus was reported [5], extending the infective spectrum of the modern exogenous bornaviruses.

With the advent of high throughput genomic sequencing technologies, various virus-like sequences buried in the host genomes are being discovered [6]. For example, the discovery of EBLs in mammalian species including humans and avian species has helped to broaden our understanding of the deep root and the flexibility at the bornavirus-host interface [7,8]. Different bornavirus-like elements (EBLs) such as EBLNs (endogenous bornavirus-like N elements), EBLMs, EBLGs, and EBLLs have been reported sporadically distributed in some vertebrate genomes [9,10]. Interestingly, some primate EBLNs have retained an intact open reading frame (ORF) [8], although natural selection has not been detected [11].

The order Mononegavirales also includes four other families, *Rhabodoviridae*, *Paramyxoviridae*, *Nyamiviridae and Filoviridae*, all known for containing highly lethal bat-borne zoonotic viruses such as rabies, Hendra, Nipah, Ebola and Marburg viruses [12]. Endogenous elements related to the *NP* and *VP35* genes of ebolaviruses and marburgviruses have been reported in the genomes of *Myotis* bats [8,13]; fragments of EBLNs and EBLLs were found in the genomes of some versper bats such as little brown bat (*Myotis lucifugus*) [8,9], Natterer’s bat (*M. nattereri*), David’s myotis (*M. davidii*), big brown bat (*Eptesicus fuscus*), and common pipistrelle bat (*Pipistrellus pipistrellus*) [14,15]. With regards to the discovery of EBLs in limited versper bat species, we expand the study to 10 bat genomes and attempt to recover a deep close relationship of bats and ancient exogenous bornaviruses.

## 2. Materials and Methods

### 2.1. Genomic Mining

Assemblies of 10 bat (order Chiroptera) genomes [16,17,18,19] were screened using tBLASTn (version 2.2.30) [20]: greater horseshoe bat (*Rhinolophus ferrumequinum*; abbreviation Rhf; GenBank accession number AWHA00000000.1); Indian false vampire (*Megaderma lyra*; Mel; AWHB00000000.1); straw-colored fruit bat (*Eidolon helvum*; Eih; AWHC00000000.1); black flying fox (*Pteropus alecto*; Pta; ALWS00000000.1); large flying fox (*Pteropus vampyrus*; Ptv; ABRP00000000.1) (the aforementioned species belong to suborder Yinpterochiroptera); Parnell’s mustached bat (*Pteronotus parnellii*; Ptp; AWGZ00000000.1); Brandt’s bat (*Myotis brandtii*; Myb; ANKR00000000.1); David’s myotis (Myd; ALWT00000000.1); big brown bat (Epf; ALEH00000000.1); and little brown bat (Myl; AAPE00000000.2) (Yangochiroptera). All six proteins of several exogenous bornaviruses were used as queries in genomic searching: Borna disease virus (GenBank number NC_001607.1, host human), Avian bornavirus isolate NM_06 (JN014948, cockatoo), Avian bornavirus isolate duck-89 (KJ756399, duck), Avian bornavirus isolate 062-CG (KF578398, goose), and Reptile bornavirus 1 strain 251,327 (NC_024778.1, snake). A series of cut-off values are used: e-value ≤ 0.001; query cover > 20%; and identities > 25%. A reciprocal tBLASNn (same cut-off values) searching against non-redundant (nr) database in GenBank (www.ncbi.nlm.nih.gov/genbank) was used to rule out false positives, *i.e.*, non-bornaviral sequences. For L protein mining in non-bat genomes, an online tBLASTn tool in Ensembl Genome Browser (http://www.ensembl.org/) was used, which targeted 40 mammals, with setting cut-off values as coverage > 30%, e-value < 1e^−10^, and identity > 30%.

### 2.2. Phylogenetic Analysis

Phylogenetic relationships of bornaviruses were inferred using the maximum likelihood (ML) method available in PhyML (version 3.1) [21]. SPR (subtree pruning and regrafting) branch-swapping and 1000 bootstrap replications were used to determine the robustness of each node. The ProtTest (version 2.4) [22] was used to select the best-fit model of amino acid substitution, which was LG (Le-Gascuel)+I+Γ for such data set. All sequences were aligned in MUSCLE (version 3.8.31) [23]. Due to most of the sequences were partial, we established two phylogenetic trees with the first contains sequences positioning 379–477 of L of BDV and the second 818–982 (Data S1). Same method as above was used for branch-swapping and bootstrapping and best-fit model JTT (Jones-Taylor-Thornton)+Γ was selected for both data sets. Abbreviations represent EBLLs in relevant bat hosts and refer to different viral copies in phylogenetic trees (Table 1; Table S1).

**Table 1:** Distribution of bornaviral elements in bat genomes.

Bat Species	Suborder *	Abbreviation	Accession	Contig Location	E-Value Identity Coverage	Indels **
*Rhinolophus ferrumquinum*	Yin	EBLN				
		Rhf.N1	AWHA01050524.1	4660–5799	2e^-36^, 77%, 50%	2, 8
*Megaderma lyra*	Yin	EBLN				
		Mel.N1	AWHB01421187.1	777–1623	4e^-30^, 78%, 43%	3, 5
		Mel.N2	AWHB01452047.1	298–714	2e^-07^, 40%, 29%	0, 0
*Eidolon helvum*	Yin	EBLN				
		Eih.N1	AWHC01264218.1	8841–9155	1e^-04^, 31%, 28%	0, 2
*Pteronotus parnellii*	Yang	EBLN				
		Ptp.N1	AWGZ01165285.1	928–1848	2e^-21^, 82%, 28%	0, 10
		Ptp.N2	AWGZ01398077.1	1–365	9e^-19^, 33%, 54%	1, 3
		Ptp.N3	AWGZ01350440.1	1486–1920	1e^-13^, 39%, 34%	0, 1
		EBLM				
		Ptp.M1	AWGZ01183839.1	1658–1996	2e^-13^, 76%, 35%	0, 2
		EBLL				
		Ptp.L1	AWGZ01393507.1	6559–10,179	8e^-79^, 56%, 36%	6, 14
		Ptp.L2	AWGZ01242856.1	1307–4498	8e^-32^, 43%, 41%	11, 10
*Myotis brandtii*	Yang	EBLN				
		Myb.N1	ANKR01245074.1	1445–1948	5e^-14^, 44%, 30%	0, 0
		Myb.N2	ANKR01266949.1	310–813	6e^-14^, 44%, 29%	0, 1
		Myb.N3	ANKR01225293.1	9897–10,340	3e^-11^, 38%, 30%	0, 0
		Myb.N4	ANKR01212309.1	7949–9532	2e^-09^, 28%, 33%	1, 0
		Myb.N5	ANKR01159012.1	25,939–26,232	9e^-09^, 26%, 39%	0, 0
		EBLL				
		Myb.L1	ANKR01212491.1	41,559–43,796	0.0, 43%, 44%	0, 3
		Myb.L2 ***	ANKR01204699.1	20,384–40,610	2e^-94^, 71%, 38%	21, 45
		Myb.L3	ANKR01204701.1	25–1592	2e^-56^, 28%, 32%	5, 10
		Myb.L4	ANKR01225293.1	11,214–13,539	1e^-42^, 28%, 43%	3, 1
		Myb.L5	ANKR01212492.1	1625–3124	5e^-26^, 21%, 33%	3, 0
*Myotis davidii*	Yang	EBLN				
		Myd.N1	ALWT01306233.1	118–612	3e^-15^, 43%, 33%	0, 0
		Myd.N2	ALWT01173634.1	13,634–13,882	9e^-10^, 22%, 40%	0, 0
		Myd.N3	ALWT01316296.1	13,281–13,532	2e^-08^, 22%, 42%	0, 0
		Myd.N4	ALWT01050150.1	238–657	2e^-07^, 36%, 28%	0, 0
		Myd.N5	ALWT01072958.1	7199–7483	2e^-07^, 29%, 33%	0, 1
		EBLL				
		Myd.L1	ALWT01131278.1	3393–9913	4e^-70^, 64%, 36%	10, 12
		Myd.L2	ALWT01213390.1	1747–5042	2e^-45^, 26%, 39%	4, 2
		Myd.L3	ALWT01141698.1	1537–3741	2e^-42^, 21%, 40%	3, 0
		Myd.L4	ALWT01026930.1	16,010–18,092	5e^-31^, 27%, 32%	4, 4
		Myd.L5	ALWT01098736.1	1601–3530	1e^-26^, 21%, 36%	4, 5
		Myd.L6	ALWT01174464.1	1245–2467	6e^-26^, 21%, 38%	3, 2
*Eptesicus fuscus*	Yang	EBLN				
		Epf.N1	ALEH01023837.1	24,020–31,033	1e^-12^, 44%, 36%	3, 2
		Epf.N2	ALEH01041783.1	76,615–77,178	2e^-12^, 49%, 26%	0, 0
		Epf.N3	ALEH01151776.1	9973–10,473	5e^-12^, 40%, 31%	0, 0
		Epf.N4	ALEH01014408.1	3710–4336	1e^-11^, 55%, 30%	0, 1
		Epf.N5	ALEH01011989.1	69,678–69,995	2e^-11^, 28%, 35%	0, 0
		Epf.N6	ALEH01076397.1	50,180–50,776	1e^-09^, 53%, 27%	0, 0
		Epf.N7	ALEH01137033.1	9537–9971	1e^-09^, 38%, 32%	0, 1
		Epf.N8	ALEH01007189.1	306–707	6e^-09^, 34%, 29%	0, 0
		Epf.N9	ALEH01110526.1	1324–1632	1e^-08^, 27%, 34%	0, 1
		Epf.N10	ALEH01074910.1	824–1277	4e^-08^, 38%, 31%	1, 1
		Epf.N11	ALEH01010737.1	10,343–10,882	1e^-06^, 45%, 24%	0, 1
		Epf.N12	ALEH01037465.1	14,375–14,874	2e^-06^, 42%, 26%	1, 0
		Epf.N13	ALEH01154995.1	4103–4420	2e^-05^, 28%, 26%	0, 0
		Epf.N14	ALEH01155661.1	6639–6935	4e^-05^, 26%, 28%	0, 0
		EBLG				
		Epf.G1	ALEH01011989.1	67,661–68,359	2e^-09^, 47%, 23%	0, 1
		EBLL				
		Epf.L1	ALEH01013293.1	16,047–20,804	0.0, 91%, 37%	0, 0
		Epf.L2	ALEH01059268.1	10,200–12,479	2e^-48^,23%,51%	4, 4
*Myotis lucifugus*	Yang	EBLN				
		Myl.N1	AAPE02027471.1	113,136–113,495	1e^-14^, 31%, 38%	2, 0
		Myl.N2	AAPE02012651.1	118,026–118,529	6e^-13^, 44%, 29%	0, 0
		Myl.N3	AAPE02006259.1	24,888–25,331	5e^-11^, 38%, 29%	0, 0
		Myl.N4	AAPE02054433.1	11,820–13,638	2e^-10^, 39%, 32%	2, 0
		Myl.N5	AAPE02007546.1	82,644–82,937	1e^-08^, 26%, 38%	0, 0
		EBLL				
		Myl.L1	AAPE02025596.1	570–7767	0.0, 64%, 45%	4, 5
		Myl.L2 ***	AAPE02049592.1	28,943–32,193	1e^-95^, 59%, 49%	16, 30
		Myl.L3	AAPE02020529.1	2038–3686	2e^-27^, 21%, 31%	3, 0

* Yin represents Suborder Yinpterochiroptera and Yang represents Yangochiroptera in Order Chiroptera; ** Frameshift number, stop codon number; BDV proteins as queries; *** Myb.L2 represents 5 viral elements located in different position in same contig, donating as Myb.L2.1–2.5; Myl.L2 represents Myl.L2.1, Myl.L2.2 and Myl.L2.3.

### 2.3. Molecular Dating

We employed two methods to date the age of the bat EBLs, based on the theories of (1) the vertical transmission, of which the virus entered the common host ancestor and was diverged after host speciation and (2) co-divergence where the viruses evolved along with the host. For the vertical transmission based dating, one pair of orthologous contigs contained EBLL sequences of Parnell’s mustached bat (AWGZ01242856.1) and David’s myotis (ALWT01098736.1) (Data S2) were used to estimate the age of the bat EBLs. Existence of transposons were screened by using RepeatMasker (version open-4.0.5) (http://www.repeatmasker.org/). We marked such pair of SINEs (Short Interspersed Elements) as SINE-A and -B, which fell in the tRNA family of retrotransposons. We confirmed the existence of SINE-A, -B and the bornaviral element in three other Vespertilionidae species—Brandt’s bat (GenBank number ANKR01171284.1), little brown bat (AAPE02024702.1), and big brown bat (ALEH01071206.1), but didn’t find any related elements in Yinpterochiroptera species, suggesting vertical transmission of such elements in Yangochiroptera. Notably, the EBLLs in the three contigs of Vespertilionidae species were not shown in our genomic mining due to the strictness of our cut-off values; however, their nature of bornaviruses was confirmed by using BLASTp (http://blast.be-md.ncbi.nlm.nih.gov/Blast.cgi) against the non-redundant BDV protein sequences in GenBank (www.ncbi.nlm.nih.gov/genbank). The date of viral integration was calculated as K/2r where K was the divergence of the 2 orthologous viral sequences in both bat genomes and r was the average mammalian neutral substitution rate – 2.292 × 10^−9^ per base pair per year [24].

For the co-divergence dating, phylogenetic ML trees were reconstructed by using all bat EBLLs and EBLNs and exogenous reference viruses. The same method as above was used for branch-swapping and bootstrapping. JTT+Γ was selected as the best model for both data sets. A group of bat EBLNs (see Section 3.3) showed host clock-like phylogenetic signals were submitted to time-scaled Bayesian inference using BEAST (version 1.8.1) [25], where a JTT model specifying a gamma distribution as a prior on each relative substitution rate. The time of the TMRCA (time to the most recent common ancestor) for such a data set (Data S1) was estimated under a Relaxed Clock Log Normal model, using Calibrated Yule model as the tree prior. Several calibration points with standard deviations giving a central 95% range that were roughly corresponding to the consensus estimate of bat speciation time [26] were used: Vespertilionoidea (Epf, Myl, Myb, and Myd) 50 My (million years), std (standard deviation) 3.65 My; Yangochiroptera (Ptp, Epf, Myl, Myb, and Myd) 54 My, std 3.65 My; Yinpterochiroptera (Mel and Eih) 58 My, std 3.05 My; Chiroptera (Mel, Eih, Epf, Myl, Myb, and Myd) 64 My, std 4.25 My. In total, 2 million steps were computed using MCMC (Markov chain Monte Carlo) sampling and parameters and trees were sampled every 200th step and 10% of the MCMC chain was discarded as burn-in. Tracer (version 1.6) (http://beast.bio.ed.ac.uk/Tracer) was used to visualize the computation until all parameters were converged and an effective sample size (ESS) > 200 was reached.

## 3. Results and Discussion

### 3.1. Bat Endogenous Bornaviruses

In this study, we have systematically screened 10 bat genomes aiming for the discovery of bornaviral elements and to address the long-term evolutionary relationships between the viruses and bats. We found four viral element types, EBLL, EBLN, EBLG, and EBLM, in eight of the 10 bat genomes (Table 1), of which bat EBLLs in David’s myotis and big brown bat were previously reported by genomic mining [15]. Noted that some non-bat EBLLs such as the ones in opossum (*Monodelphis domestica*), Tasmanian devil (*Sarcophius harrisii*), yellow fever mosquito (*Aedes aegypti*), and American house spider (*Parasteatoda tepidariorum*) were also reported [10,15]. Overall, EBLG and EBLM were rarely detected (with only 1 copy each), while EBLL and EBLN were more frequently found, suggesting that a low frequency of integration of EBLG and EBLM during Chiropteran speciation. Bats in the suborder Yinpterochiroptera harbored low (*n* ≤ 2) or no EBL copies (Table 1): only one copy of EBLN in the straw-coloured fruit bat, one in the greater horseshoe bat and two in the Indian false vampire. In contrast, the EBLs in Yangochiroptera species were more robust in terms of viral copy numbers (6 ≤ *n* ≤ 17) and element types (*n* = 4). Such observations indicated that bornavirus-bat interaction was relatively more active in Yangochiroptera or reverse-transcriptase activities of transposable elements within genome after its divergence with Yinpterochiroptera.

### 3.2. Intact Bat EBLL

**Figure 1:**
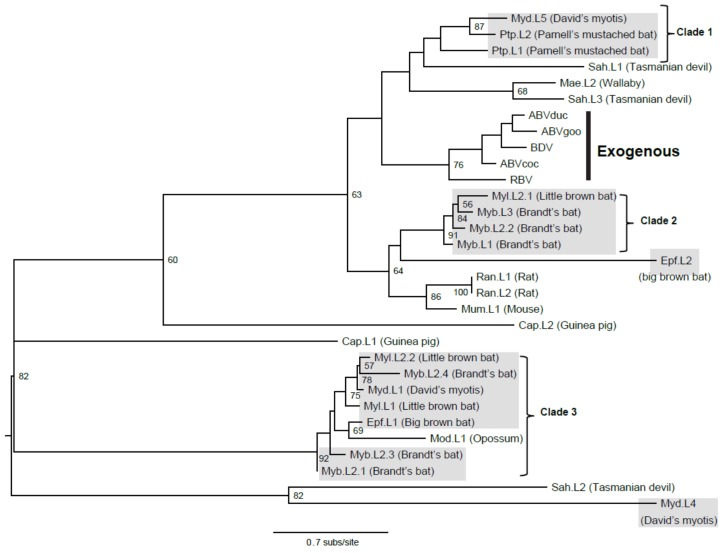
Phylogenetic positions of bat endogenous bornaviruses. EBLL protein sequences of bats and non-bats are used to construct the phylogenetic tree. Host names indicate viral lineages; the numbers denote viral elements in different contigs and the sub-numbers denote different viral elements in same contigs. The abbreviations can be found in Table 1 and Table S1. Exogenous bornaviruses are highlighted; all bat clades are marked. Bootstrap values lower than 50% are not shown. Branch lengths are drawn to a scale of amino acid substitutions per site (subs/site). The trees are midpoint rooted for purposes of clarity only. All bat EBLs are shaded in gray.

Previous study showed that EBLN was intact and expressed in some primates such as humans [8], however we didn’t find such pattern in any of the 10 bat genomes. Strikingly, however, we found that the big brown bat has maintained a nearly complete L protein sequence with no stop codons observed at the nucleotide level (accession number ALEH01013293.1; 93.3% coverage of the BDV L; Figure S1) in its genome. Such an intact bat viral element was also reported at a recent international conference and designated as efEBLL-1 (https://myiums2014.zerista.com/event/member/125781). We then tested the maintenance of L (using the newly found bat EBLL as well as other bornavirus L proteins in Section 2.1 as query) in non-bat species and only revealed three rodents and three marsupials buried similar viral elements (Table S1), with short protein sequences and no intact forms, indicative of the infrequent infiltration of EBLLs in non-bat mammals. Such bat EBLL didn’t fall into the phylogeny of exogenous BDVs (Figure 1; Figure S2), suggesting such virus was highly diverged compared to their counterparts in birds and mammals.

### 3.3. Viral Transmission

In EBLL trees, bat viruses occupied at least four major diversified phylogenetic positions/clades (Figure 1; Figure S2), indicative of multiple bornaviral invasions into bat genomes. Within the same clade mentioned above, a phylogenetic incongruence of viruses from versper bats (genera *Myotis* and *Eptesicus*) was observed, indicative of a cross-species transmission occurrence. Frequent bornaviral invasions were commonly found in Yangochiroptera species (including *Myotis* and *Eptesicus*) for EBLLs and EBLNs (Figure S3). Some EBLNs were even closely distributed within same contigs (Figure S3B), indicative of either frequent invasions of the similar viruses into the versper bat genomes or small-scale segmental duplication near the viral integration sites. Previous study suggested that LINEs-1 (long interspersed nuclear elements-1) could facilitate integration of EBLs [15]. Importantly, loss of LINE-1 activity in the megabats has been documented compared to microbats [27], which was in line with our observation of the absence of EBLLs and infrequence of EBLNs in megabats. Moreover, versper bats could have undergone several waves of such activity in their genomes; however, this remains to be confirmed.

### 3.4. Molecular Dating

**Figure 2:**
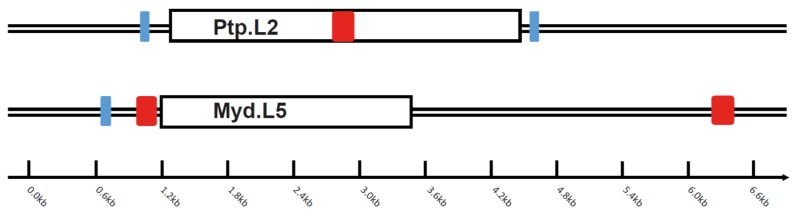
A pair of orthologous viral contigs in Parnell’s mustached bat and Brandt’s bat. The boxes with position 1.3–4.5 kb in Ptp.L2 and 1.2–3.5 kb in Myb.L5 represent viral element regions. The same color (blue and red) represents orthologous SINEs, where SINE-A1 and A2 are duplicates within Ptp and SINE-B1 and B2 are duplicates within Myd. The bar represents the contig length (in kilobase).

We attempted to evaluate the age of the ancient exogenous bat bornaviruses by employing two different methods. First, we found a pair of SINEs, with one EBL sandwiched, shared by two distantly related bat species—Parnell’s mustached bat and David’s myotis (Figure 2). The lack of such viral elements in other *Myotis* species and *Eptesicus* was probably due to purging of non-self genomic sequences during host evolution. We confirmed such a viral element (termed Ptp.L2 in Parnell’s mustached bat and Myd.L5 in David’s myotis) inserted into the bat ancestor, by perfectly aligning the flanking (non-viral) regions of the EBL between the two species. Because an artifical cut-off was set in advance, the flanking regions could contain highly diverged viral sequences that have not been detected *in silico*. We also confirmed the orthologous relationships of SINE-As and -Bs by using aligning method (Data S2). The pairwise genetic distances (divergence) of these SINEs were calculated measuring by using *p*-distance with pairwise deletion in MEGA (version 6.06) [28]: 0.172–0.175 for SINE-As and 0.295–0.336 for SINE-Bs. The two aligned contigs in Parnell’s mustached bat and David’s myotis differed by 22.1% (retrotransposons were removed) of the nucleotides (Data S3). We thus estimated the common ancestor of the bat EBLs to at least 50 My old, margining to the divergence time of the two bat species at ~55 My ago.

**Figure 3:**
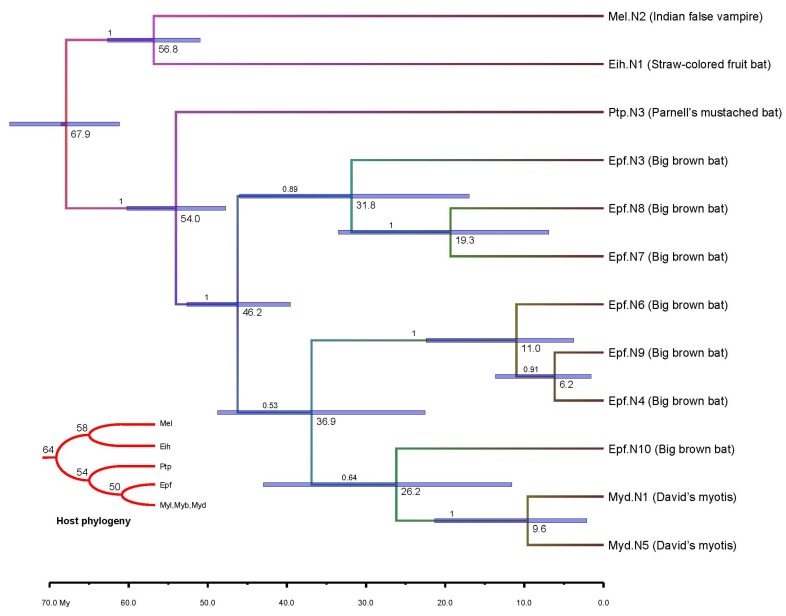
Time-scaled bat bornavirus evolution. The abbreviations of bat hosts represent bat EBLNs. A host species tree, with divergence time (My) marked, is embedded to highlight the virus-host co-divergence. Key nodes represent ages (My) of root-to-tip and 95% credible intervals are shown by horizontal bar. Bayesian posterior probabilities are given on the branches.

Second, we found that one group of bat EBLNs (*n* = 32) exhibited host clock-like topology in maximum likelihood (ML) phylogeny (Figure S3B), suggesting a co-divergence of the viruses and hosts. We then ruled out the vertically transmitted viral elements by checking the flanking regions of all viral elements. We applied the rationale that if vertical transmission occurred in the common ancestor of several different species, the viral elements from those species were excluded; if several viral elements within a single species shared a common ancestor (*i.e.*, formed by within-genome duplication), we reduced them to only one representative viral element. Such analysis reduced the viral numbers to *n* = 12. We then applied Bayesian inference to confirm the phylogenetic topology and more importantly yielded the ages of different bat bornaviruses (Figure 3). Such molecular dating exhibited a continuing interaction of bats and bornaviruses started from the origin of bats (71.1 My ago) to the present time as seen in versper bats (Figure 2). The younger ages of most versper bat viruses, together with the frequent invasion, clearly inferred the likelihood of modern exogenous bornaviruses could be circulating in these bat species.

## 4. Conclusions

Previous genomic mining revealed eukaryote species involving mammals, reptiles, fishes, insect and spider have been identified to have EBLs in their genomes [8,9,10,15]. Due to limitation of reported bat EBLs—for example, only versper bats in genera *Myotis* and *Eptesicus* were found to have such viral elements [9,10,15]—we expanded our genomic mining toward the ten available bat genomes. Overall, we delineated the deep root of bat bornaviruses and the evolutionary relationships of EBLs and their bat hosts. Several viral infiltration patterns were established, such as viral insertion in ancestral bats, inert integration in Yangochiroptera species, frequent invasions in versper bats, and virus-host co-divergence. We showed that EBLL infiltration was robust in bats compared to non-bat species and bornaviral invasion is likely occurring in some versper bats such as the big brown bat. It was reported that some of the EBLNs are still being expressed as mRNAs in some hosts and expressed proteins could interact with host factors and function as inhibition of viral replication [29]. Our data show a long-term bond of versper bats and bornaviruses and argue strongly for a more targeted and systematic bornavirus hunting in bat populations.

## Supplementary Files

Supplementary File 1
