# Supplementary material for: Genomic Mining Reveals Deep Evolutionary Relationships between Bornaviruses and Bats
Source: Viruses. 2015 Nov 10;7(11):5792–800. doi: 10.3390/v7112906 (PMC4664979; doi:10.3390/v7112906)
Supplement: Supplementary File 1 [file viruses-07-02906-s001.pdf]

viruses

MDPI

Supplementary Materials

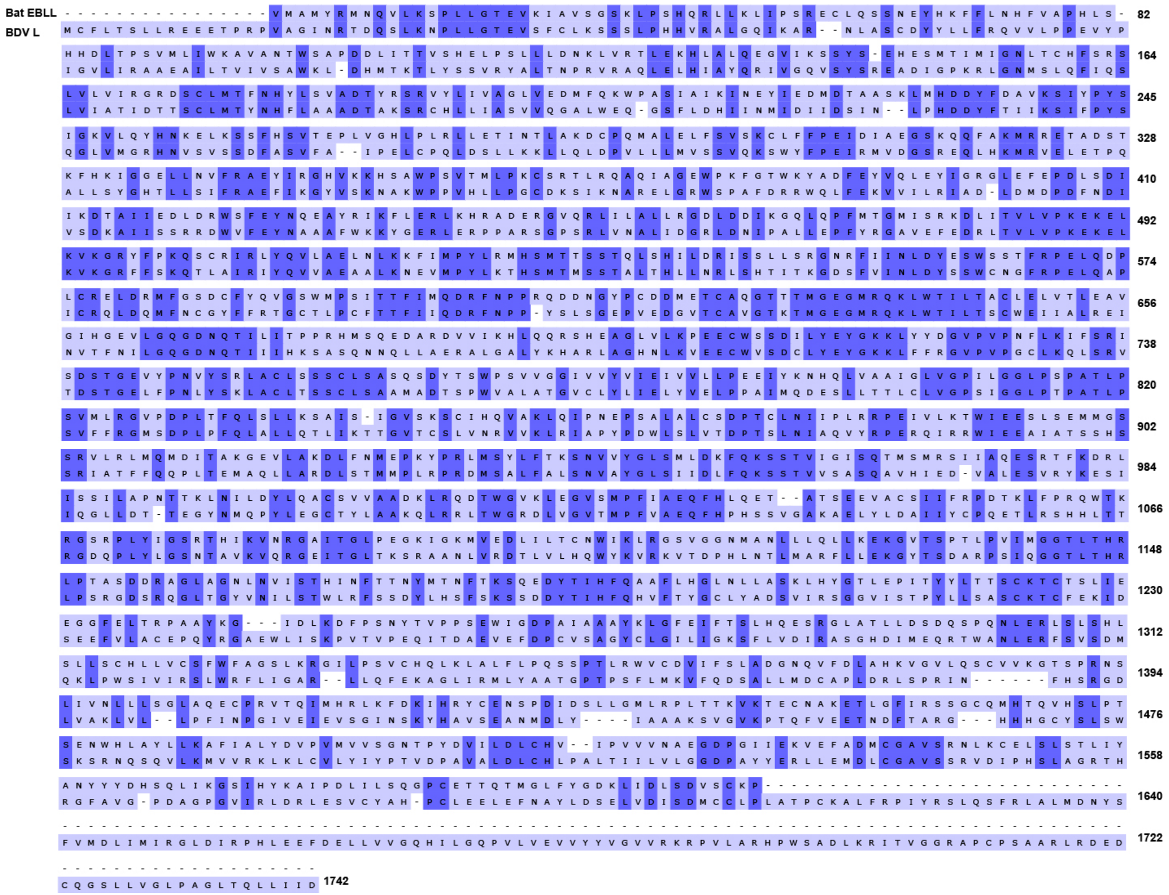

Figure S1. Alignment of the nearly complete bat EBLL and BDV L. The positions refer to the complete human BDV L protein sequence. The conserved amino acids are highlighted using dark blue.

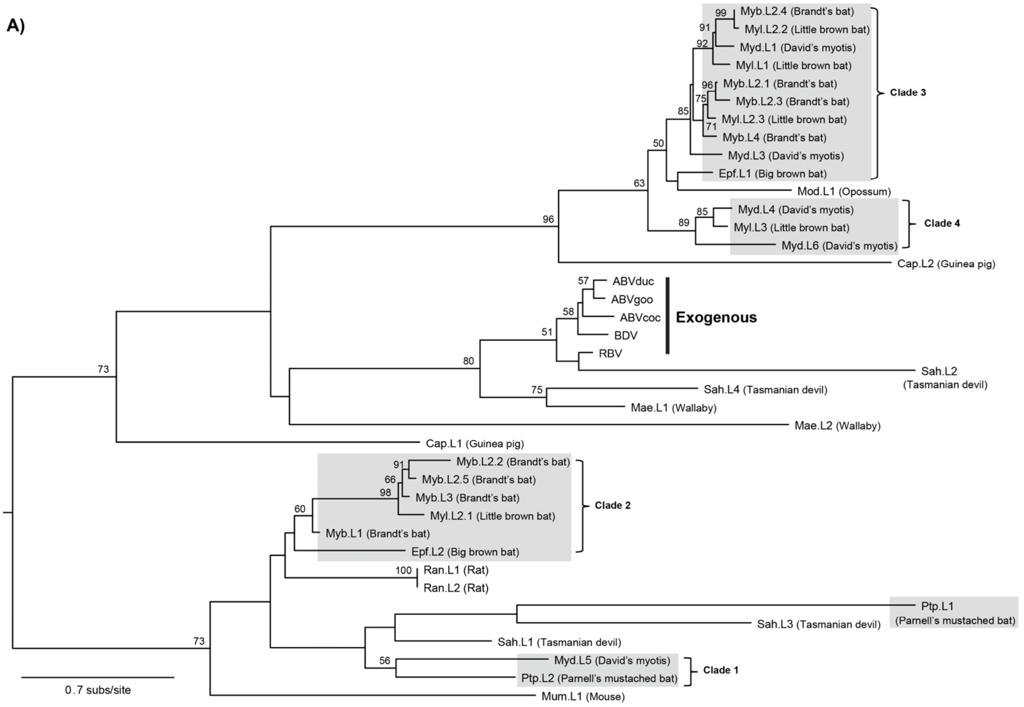

Figure S2. *Cont.*

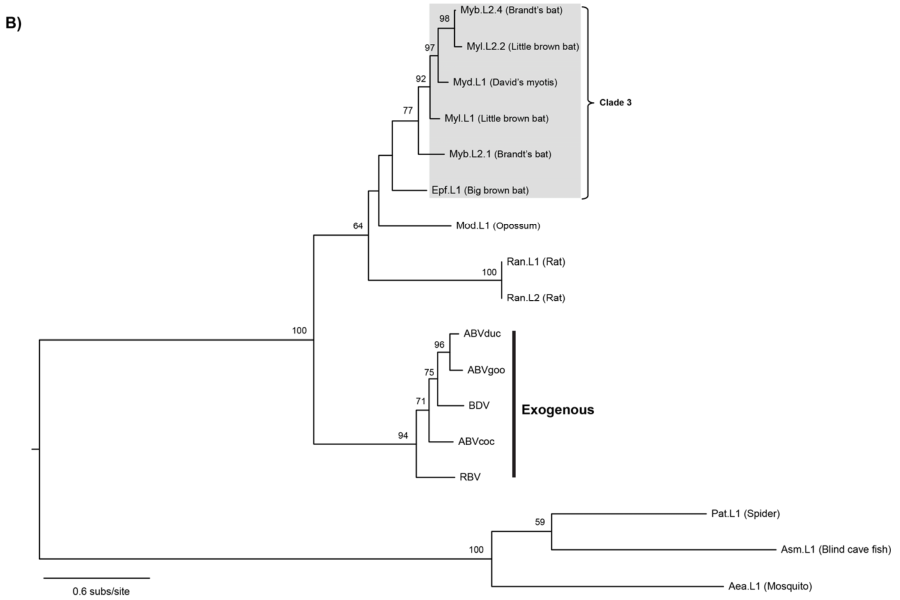

Figure S2. Phylogenetic positions of bat endogenous bornaviruses. Phylogenetic relationships of bat and non-bat EBLLs, and (**A**) are mammalian EBLLs and (**B**) includes three non-mammalian EBLLs. The abbreviations of hosts, given in Table 1 and Table S1, are used to denote viral lineages; the numbers denote viral elements in different contig and the sub-numbers denote different viral elements in same contig. Bootstrap values lower than 50% are not shown. Branch lengths are drawn to a scale of amino acid substitutions per site (subs/site). The trees are midpoint rooted for purposes of clarity only. All bat EBLs are shaded in gray.

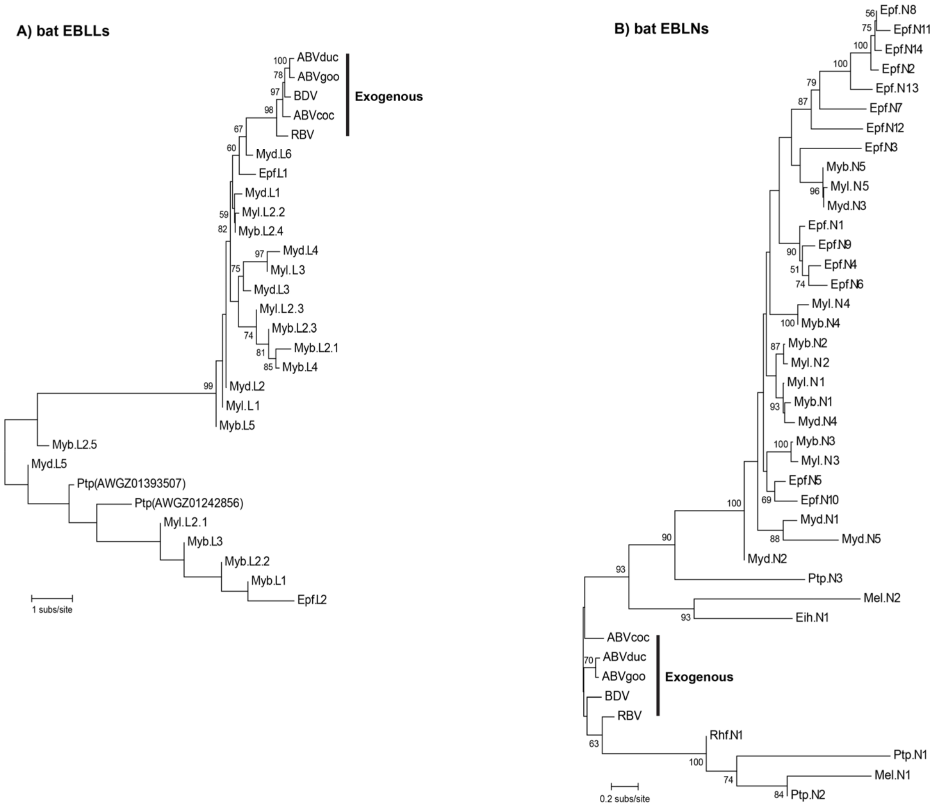

Figure S3. Phylogenetic trees of bat (**A**) EBLLs and (**B**) EBLNs. The abbreviations of bat hosts, with contig numbers, given in Table S1, are used to denote viral lineages; the numbers denote different viral elements in same contig. Bootstrap values lower than 50% are not shown. Branch lengths are drawn to a scale of amino acid substitutions per site (subs/site). The trees are midpoint rooted for purposes of clarity only.

Table S1. Genomic mining of EBLLs in non-bat mammalian genomes.

Species Name	Common Name	Order	Class/Infraclass	Genomic Location *	Abbreviation **
Cavia porcellus	Guinea pig	Rodentia	Mammalia/Placentalia	scaffold_10:39865026-3986598	Cap.L1
				scaffold_24:23357547-23360406	Cap.L2
Macropus eugenii	Wallaby	Diprotodontia	Mammalia/Marsupialia	GeneScaffold_790:272034-272628	Mae.L1
				Scaffold25161:23630-24772	Mae.L2
Monodelphis domestica	Opossum	Didelphimorphia	Mammalia/Marsupialia	6:184038144-184036177	Mod.L1
Mus musculus	Mouse	Rodentia	Mammalia/Placentalia	19:30533881-30536906	Mum.L1
Rattus norvegicus	Rat	Rodentia	Mammalia/Placentalia	1:256122156-256126149	Ran.L1
Sarcophius harrisii	Tasmanian devil	Dasyuromorphia	Mammalia/Marsupialia	GL849720.1:322916-325367	Sah.L1
				GL841178.1:56153-58900	Sah.L2
				GL856748.1:1817214-1818286	Sah.L3
				GL857007.1:960360-961142	Sah.L4
Astyanax mexicanus	Blind cave fish	Characiformes	Actinopterygii/Neopterygii	APWO01063603.1: 2029-3147	Asm.L1
Aedes aegypti	Yellow fever mosquito	Diptera	Insecta/Neoptera	AAGE02002824.1:10733-11932	Aea.L1
Parasteatoda tepidariorum	American house spider	Araneae	Arachnida	AOMJ01143983.1:434-1690	Pat.L1

* Chromosome/scaffold/contig:location in Ensembl or GenBank; ** Abbreviations represent EBLLs in relevant non-bat hosts and refer to different viral copies in phylogenetic trees.

Data S1. EBLs alignments (phylip format) used for phylogenetic analyses.

1) The first dataset of EBLLs, covering position 379-477 of L of BDV.

```
   31    109
BDV        QLFEKVVILR IADLDMDPDF NDIVSDKAII SSR-RDWVFE --YNAAAFWK
ABVcoc     KWFKDVIILK VSDLDLDPDF NDIVSDKAVI NSK-RDWVFE --YNAAAYRH
ABVduc     KLFSEVVILK IADLDLDPDF NDIVSDKAII NSR-KDWTFE --YNAAAYRR
ABVgoo     KLFTEVVILK VTDLDLDPDF NDIVSDKAVI NSR-RDWTFE --YNAAAYRK
RBV        HWFRYVSIQK VFELDTDPDF NDIVTDKAVI ESR-ASWPFE --FNSAAHRH
Epf.L1     SSRVLRLMQM DITAKGEVLA KDLFNMEPKY PRL-MSYLFT --KSNVVYGL
Myb.L2.1   SSRILKLLQM DITSKGEMLA NELFNMEPKY PRL-LSYIFT --KSNAAYGL
Myb.L3     HFSEVCLEVA GKRLQFEPDL SDIVTDRAII ENH-K-WNFE --YNAEVY--
Myd.L1     SSRVLKLMQM DITSD-ETLT NGLFTMGPKY PWL-MSYLFT --KSNVAYGL
Epf.L2     DFKYVQLEFS GHRLECEPDL SSIVTDKAVT EDR-NRWNYE --DNAEAYSE
Myb.L1     DFSEVCLEFA RKGLEFEPDL SDIVTDKAII ENR-NKWTFE --YNTEAYFN
Myb.L2.2   HFSEVCHEFA GKELESEPDL SDIVTDKAIS ENH-NKWTFE --HNTEVY--
Myb.L2.3   SSRILKLLQM DITSKGEILA NDLFNMEPKY PRL-MSYLFT --KSNAAYGL
Myb.L2.4   SSRFLKLMQM DITSEGETLA NGLFTMEPKY PCV-RSYLFT --KSSGAYGR
Myd.L4     CSTGCSYYRK RYEIKIMDNY DNCL--EALV LGE-LGFNGD --IMGQGDNQ
Myd.L5     DFQDISLDAS YIPLDLNPDI NNLVSPKSIT ESR-THWTFD --FNIVAYRE
Myl.L1     SSRILKLKQM AITSKGETLA NHLFTMEPKY PRS-MSYLFT --KSSIAYGL
Myl.L2.1   NFSEVCLEVA GKGLQFQPDL SDIVTDKAII ENH-TSGPFQ ----------
Myl.L2.2   SSRFLKLMQM DITSEGETFA NSLFPMEPKY P--------- ----------
Ptp.L1     ---------K VYNLDLNPDI NDLVSDKSII E-R-KHWAYE --LDVTAYRE
Ptp.L2     DFQDVSLDTS STSLDLNPDI NDLLSDTLII ESR-KHWKYE --FNIVAYRE
Mum.L1     AFRHVQLLAA VNQLKYE--M RDIVTDKAII EDG-SKWAFE --PNSEAYRK
Mod.L1     SPKVLWLMQM DITTKGKIIA DDLYSMDPPF PRL-MSYIFN --KLNVAYGL
Cap.L1     ------KILY GTPLKYQLTT KEWC---AIV PKRSKQFVLV --KKLQRPGI
Cap.L2     DTYGLNIVTR -------PFI AEQFLSNTMT PER-----FV -----AVYNP
Ran.L1     DFEHAQLPAA DNQLEYEPDL RDIVTDKAVL EDG-NKWTLE --HNSDIYRK
Ran.L2     DFEHAQLPAA DNQLEYEPDL RDIVTDKAVL EDG-NKWTLE --HNSDIYRK
Sah.L1     -EFEDVQLLE TKPLDLNATI NDFDSDKSVI DTR-EHWT-K --NIIQSTGK
Sah.L2     -------HAL CKELDMFLAN GQFYQAESII LKY-TTFIVD KFYSSQSDDL
Mae.L2     -AFSIVSLFG DNIFHCDPYT SELVSDKSI- -TK-KKYAYG --YNI---YL
Sah.L3     DFSALSLLQG DNVF-YDPHI SKLISDKSII ERD-KK---- ----------

           KYGER--LER PPARSGPSRL VNA---LIDG RLDNIPALLE PFYR--GAVE
           KHGHK--LER PPERSGPSRL VNA---LIDG KLDSVPELLE PFYR--GSVA
           KYGEQ--FER PRNRSGPSRL VNA---LIDG RLDDIPGLLL PFYR--GAVE
           KHGDQ--LNR PEKKSGPSRL VNA---LIDG RLDDIPTLLI PFYK--GSVE
           KHKKP--LDR PTGGKGVSRL VNA---LIDG KLDNIPKLLE PYMV--GSVD
           SMLDK--FQK SSTVIGISQT MSMRSIIAQE SRTFKDRLIS SILA--PNTT
           FTLDK--FQK SSTVIGISQS MSMRS-ITEG SKRFKDQLIR SILA--PNTR
           -YHQR--LRH IDRSQGLQQL ILA---LLRG ELDDIKKRVE PFIT--RTLC
           SMLDK--FQK SSTVIGISQN MSMKS-ITEE SKRFRDQLVR SILA--LNTK
           AQKDDSAFNE RVSVINPCPL SRK---LGQP RRENGALFVG KACR--PHYR
           KYHQR--LRH IDKSQGL-QL ILT---LLSG ELDDIKKRIE LFMT--GTLC
           -YHQR--LRH INRSQGLQQ- ILA---LLSG ELDDIKKRVE PFLT--RTLC
           SMLDK--FQK SSTVIGISSM SMRC--ITED SKRFKDRLIR SILA--PNTR
           SMLVS--FQK SSTVIGISQN MSM------- --KSITEESK RF----RNTK
           TLIVK--TPL TMTKEECKKK LLE---ILES RALEAGSVLK PE------EC
           KHKQP--KQH L-AKHGLKCL VKA---LVDG HLDNVPAMLV PFAQ--G---
           SMLGK--FQK SSTVIGISQT MSMKS-ITEE SKRFRDQLVR SILA--PNTR
           -YHQI--LRH IHRSQGLQQL ILA---LLSG -LDDIKKRVE TFPR--GTLC
           -------FQK PSTVIGISQN MSMKS-ITEE SKRFRDHLVR SILA--LNTK
           K-QQL--LWY P-APHGLKHL VTA---LVDG YLDNVPTLLA LFCP--GELN
           KNKQP--IQH P-------RF VKA---LVDG HLVNVPAMLA PFCV--GKLK
           KHSQR--MHL IDSKGGV-RL IPA---LLAG NLDNIREKVK PFCT--GVLN
           TMINK--FQK SSTVITISQS MMMRS-LTLA SRNFLFQVIN SIMR--PNLS
           LSSNR--LIN INVKIA-SRY GDS---AIAT EFKLGNKIFN SLHL--DARK
           VIGYS-CLTR IEDMYDQQRC YYCTT-LSER QMVAAQTALC DWTKSPGVVD
           KHGQR--PWY TDSNRGM-WL VLV---LLAG SLDN---KVK PFST--EMLN
           KHGQR--PWY TDSNRGM-WL VLV---LLAG SLDN---KVK PFST--EMLN
           SLKIS--LRY P-LNHGVKRL VKA---LIGD LLDDTLIILE LYCF--GQID
           PIEDK---RT CIHGTGSSRK GMQ---RLWT IMSSCSELIV PEDD--GISL
           EYQKP--IHL P-ESKGTKIL IEA---LIDG KLENIISILT LHCT--GELE
```

---------- --------QL VKA---LNDG KFDNIVGILT LHCT--GELE

FEDRLTVLV
FDDRITVLV
FDDRITVLV
LDDRITVLV
IRDRITILV
KLNILDYLQ
GLNIIDYLT
AHDLITVLE
GINRMDYLT
ASAKA----
AHDLITVLV
AHDLITVLV
GLNIIDYLS
GINRMDYLT
GLNILDFLH

GINIMDYT-
AHNLITVLV
GINRMDYLT
SE--ITVLT
SGMCFTVLV
-QDLIVVLV
GINIFDYVE
PNTTPDFWY
SKSTNKLFD
SQDLIIVLV
SQDLIIVLV
MNHYLTVLV
GSIPQLLLL
GQNLLPVLV
G-NLLPLLD

2) The second data set of EBLLs, covering position 818-982 of L of BDV.

39 179
BDV SDPLPFQLAL LQTLIKTTGV TCSLVNRVVK LRIA-PYPDW LSLVT-DPTS
ABVcoc SDPLPLQLAL LKTLLRLTGV SIEFVNSVVK LKIA-AYPDW LSLIT-DPTS
ABVduc SDPLPLQLAL LQTLISTTSI SSSFVNRVVK LTIA-AYPDW LSLVT-DPTS
ABVgoo SDPLPLQSAL LKTLVSLTSI SISFVNRVIK LKIA-AYPDW LSLVT-DPTS
RBV SDPLPLQLAL LKEALNSTNV SLSLINRVVK LKIS-SSTDW LALVT-DPTS
Epf.L1 SMPFIAEQFH LQETATSEEV ACSIIFRPDT KLFP---RQW TKRGS-RPLY
Epf.L2 ERLQVAGRYF SKQS-----C NIRLYQVLAE PNLKEFVMPF IRMHS-MTAS
Myb.L1 KELKVKGRYF LKQS-----C SIRLYQVLAE LNLEKFVMPY LRMHSMTTSS
Myb.L2.1 TMPFIAEQFA LRETVPELEV AQS-IFRPDN HLFP---REW MVRGK-HPLY
Myb.L2.2 KELKVKGRYF SKQS-----C TISFLKVQFC KNLI-FIMPY LWMHSMTTSS
Myb.L2.3 TMLFIAEQFA LRETVPELEF AQS-IFRPDN HLFP----RE MVRGK-HPLY
Myb.L2.4 TMPFVAEQFI MQETVSRCD- --SIIFRPDE L----FPRLS MIRGK-RPLY
Myb.L2.5 ------GRYF SKQS-----C TISFLKVQFC KNLI-FIMPY LWMHSMTTSS
Myb.L3 KELKVKGRYF LKQS-----C TISFLKVQFC KNLI-FIMPY LMHSM-TTSS
Myb.L4 TMPFIAEQFA LRETVSELEV A-SIIFRTDK DLFP---REW MVRGK-RPLY
Myd.L1 TMPFVAEQFI MQESMCEADV SKSIIFRPDE ELFP---RLW MIWGK-RPLY
Myd.L3 TMPFVAAQFV LQLTVSEPEV ARSIIFCPDH SLLP---REW ITRGK-QPLY
Myd.L4 TMPFIAEQF- ---------- ---------- ---------- ----------
Myd.L5 ------GKYF SVQSLHHVYR GSR---DEYK KTHHATNHCL MTMTS-TQLN
Myd.L6 TMPSIAEQFL CNSAIAQS-V RRSIIFRPES LLEP---RCW VKRGN-KSLH
Myl.L1 TMPFVAEQFI MQEIVSEGDV SKSIIFRPDE ELFP---RLW MIRG--RPLY
Myl.L2.1 KELTVKGRFS KQS------C TIRFLKVQFC KNLI-FIMPS LQMHSMTTSS
Myl.L2.2 TMPFVAEQFI MQETVSRCD- --SIIFRPDE L----FPRLS MIRGK-RPLY
Myl.L2.3 TMLFIAEQFA LRETVSELEV AQS-IFQPDN DLFP---REW MVRGK-HPLY
Myl.L3 TMPFIAEQFQ LCNSATSQSV RRSIIFRPAS LLEP---RCW VKRGN----R
Ptp.L1 --LQQKGRYS VQSLNPRVFL FVAVMNIKDH VMLS-IETHS MTVTS-TKLN
Ptp.L2 KELKRKARFF SVQSTLASVS QKKLHHQVYQ VVAEIYIKEC VMPL----IQ
Mum.L1 RELTVKGRHS PRQS-----Q SIHLYQVLAE LNLKKHSMPS QVTFCEKFIY
Mod.L1 IKPFIAEQFK LRESAREDDI ADSIIFRPED EILP---RD- THRGS-RPLY
Cap.L1 SSPTLKALFS LHDD------ --QTFDLACK LSVPPYVPLY LSYGG-TTVC

Cap.L2 TFTSHAGRHF NPQTTNSQSW ADRSLKVIST HVMF-TTHFM TDFGKSKDYT
Ran.L1 RELKVKGRYF LKQL------ ----YQVLAE LNLR----NI LCPLRSHSIK
Ran.L2 RELKVKGRYF LKQL------ ----YQVLAE LNLR----NI LCPLRSHSIK
Sah.L1 -MLKRKDKYF SVQS-----L SNQVYQIVSE MNIKNWIMSY IQTHTLTKMS
Sah.L2 EDPLPFQFAV ITAI---KEI PLNIVDQLRK IKVS-YSKRF IALNS-YSIC
Sah.L3 KELNRKGLFF SVQI-----L EKRIYLSCDQ HKEY-IVPNI ITHIIMSPTR
Sah.L4 SDPLTYKLCA LKAGASQYIP PLLLLNEVIT LPTV-FETGF LVM---EPTS
Mae.L1 SDPLKYQLHV LRIPVSQQKP PL-LLNQVIT LTLSNNVHDW MLLVM-EPTS
Mae.L2 ASSLVCRLW- KRESIKLLVR SLEAHNALNN I-------KW SATRL-HHAF

LNIAQVY--- ------RPER QIRRWI---E EAIATSSHSS RIATFFQQPL
LNIQQLL--- ------RPER QIRKWV---E QAILASSHSS RVATFFQQPL
LNIAQVF--- ------RPER QIRKWV---E EAISASSHSS RVGEFFQQPL
LNIAQVL--- ------RPER QIRKWV---E EAIAASHHSS RVGEFFQQPL
LNISQLL--- ------RPER QLRRWV---E QAINQSTHSS RVADFFRQPL
IGSRTHIKVN RGAITGLPEG KIGKMV---E DLILTCNWIK LRGSV-GGNM
ATQLSHV--- ----LDKIAQ QITSQN---A LIVLNLDYES WCDAF-WPEL
TQLSHIL--- ---------D KTSAYITRKN SFVVNLDYES WCNAF-CPEL
IGSRTFVTIN RGAITGLPEG PIGKMA---E DLISTCDWIK IKGAG-GQHL
TKLSHIL--D KKYQHRLPEK ---------N SLVVNLDYKS WCNAV-HPEL
IGSTFVT-IN RGAIMGLPEG PIGKMA---E DLISTCDWIK IKGAV-GQHL
IGSRTFAKIN REAITGLPEG RVGKMA---Q DLISTCDWIK MKGAF-GQHL
TKLSHIL--- -----DKFLT KISAIT-RKN SFVVNLDYKS WCNAV-HHEL
TQLSQIL--- --------DK KISAYISRKN SFVVNLDYKS WCNAV-HPEL
IGSRTFVKIN RGAITGLPEG HIGKMA---E DLISICDWIK IKGAV-GQNL
IGSRTFVKIN RGAITGLPEG RIGKMA---E DLIPTCDWIK IKSAF-GQHL
IVSRTFVKIN RGAITGLPEG RIGKMA---K DVISTYDWIK IKGTV-GQNL
IGSRTFVKIN QKAIMDLPEG KIGKAA---E NLVATCGWIK LHRSV-GPDS
NALEKLP--- ---------- HVARRQ---D KFVIKLDYSS WCNYF-RPEV
IGSRTFVKIK QEAITGLPEG NLGKMA---E DLTAACDWIK LLRSV-GPNL
IGVKNICKIN RGAITGLPEG LIGKMA---- DLISTCDWIK IKGAV-GQHL
TQLSQIL--- --------DK KISAYITRKN SFVVNLDYKS WCNAV-HPEL
IGSRTFANIN REAITGLPEG QVGKMA---Q DLISTCDWIK MKGAF-GQHL
IGSRTFVTIN RGAITGLPEG RTGKMA---E GLISTCDWIK IKGAV-GQHL
IGSRTFVKIN RKAITGLPEG KIGKTA---K DLVATCDWIK LHRSV-GPNS
HTLAKFS--- --HVATFYDK FIINWT---V HLGSAISDLR PNIYC-VQNW
MQLNLVL--- ------AKLS HIATIL---N KFVINLDSSS WCNYF--PEI
SAISQFR--- -HDLHESNTR LLVCCK---P GLVMVCSLPR VT----APAW
IGSRTFVKIN RGCL----KV NLGKMA---- DIVPACEWIK LQGEVLNGNL
LGVSPNI--- -----RPARE EIKTWHSAKE ELGVPGSRLD WH--------
FPFAAFIKIN RGAITALRVK R-GKWQ---P RQLCVIGLNH L-----VLTL
TSSTELS--- ------HNSH KISTRIVQDG TFVVNLDYES CS----PPRV
TSSTELS--- ------HNSH KISTRIVQDG TFVVNLDYES CS----PPRV
VRLNNAL--- ------AKLA NVSTDH---T KFVINLGYSS -CSYF-RPEV
LKMSVTE--- ---------- NRTSAQ---R CYVNRCVYKV ASIVF-NIFN
LHHELYP--- ------LANQ FICQNK---I CGQETISYDA NTSYL---NR
LNIKYEL--- ------KPES VLRKVK---D ATLTASSF-- -LAYFFKVPL
PNVKHEL--- ------RPER VLRKWA---E DEISMSTASL SLANFFKRPL
MELTFGV--- ---------- ---------- RFVANFDY-S LCKDF----I

TEMAQLLARD LSTMMPLRPR DMSALFALSN VAYGLSIIDL FQKSSTVVSA
TDMAQILARD LSSMMPLRPR DMSALFSLSN VAYGLSIIDL FQKSSTVVSA
TEMAQLLARD LSTMMPLRPR DMSALFGLSN VAYGLSVIDL FQKSSTVVAA
TEMAQLLARD LSTMMPLRPR DMSALFSLSN VAYGLSVIDL FQKSSTVIAA
TELAQMLAYD LSTMIPLRPR DMSALFGLSN VSYGLGIIDL FQKSSTVISA
ANLLLQLLKE KGVTSPTLP- ---------- VIMGGTLTHR LPTASDDRAG
QDPICAELDR LFQSECFFQV GSWMP-AVTT FITQGRVNPP EPGDAGLPGE
QGPLCEELDR LLGSGCFFQV GSWMPV-LTT FIIQDRFNPP AQRDNGLPKE
ARLLSILLSE KGVHTPDLP- ---------- VIMGGTLTHR LPTASDDRPE
QGPLCEELHP TKKHPD--PR SSWMPLLTTF II--QDFNPP AHRDDGLPKG
ARLLSILLSE KGVHTPDLP- ---------- VIMGGTLTHR LPTASDDRPE
ATLLSTLLSE KGVHPQELP- ---------- VIMAGTLTHR LPTASDDRAG
QGPLCEELHP TKKHPD--PR SSWMPLLTTF II--QDFNPP AHRDDGLPKG
QGPLCEELHP TKKHPD--PR SSWMPLLTTF II--QDFNPP AHRDDGLPKG
AILLSILLSE KGVHIPDLP- ---------- VIMGGTLTHR LP--SDDQAE

AMLFSTLLSE KGVHAPEFP- ---------- VIMAGTLTQR LPTASDDRGG
AQLLSVLLSE KSVHVPNLP- ---------- IIMGGTLTHR LPTASDDRAG
KQLLSCLLEE KDVIPPDDP- ---------- AVLGGTLTHW LPTASDDRSG
YALGAEM--D VLGCGEFFKT GNLTLRYTMS FIIQDKFNPP CQLPDRLPME
KQLLSCLLED KGMIPPDVPS VGWM---LTT RCQQLRMTDL YSLGPTSCQP
ATLLSTLVSE KGVHAPELP- ---------- VIMGGTLTHR LPTASDARAG
QGPLCEELHP TKKHPD--PR SSWMPLLTTF IVQDF----- ----------
ATL-STLLSE KGVHPQELP- ---------- VIMAGTLTHR LPTASDDRAG
ARLLSILLSE KGVHTPDPP- ---------- VIMGGTLTHR LPTASDDRAG
KQLLSCLLEE KDVIPADDP- ---------- AVLGRTLTHP ----SDDRSE
TYFLDVDGSS --------K- ---------- --LEACFGTL HSSNRTDKFN
QYALCAESDV FLGCDKFFRT G-NIMLRYTT FTTQDKFNLP WQLPDGLPVE
SGHWCISLSR LFVSGGFLGT GSHGIYNPGV LSKRRQYSPH PPAWGNLLSR
AELFTTLLKE KSITPPSLL- ---------- VVIDGTLNHR LPTSSDDARI
TRTKSCQNHN LY------PS LIAGPHPHLT MLLK-TYIAL YQG-------
KALINCLIKE EVSLPDL-P- ---------- VMLGGTLTHR LP--TVDRAG
TALPDQGFDI LFSVICFFQV GVWVLV-LTT FIIQDRFMSP KQGDNILPTE
TALPDQGFDI LFSVICFFQV GVWVLV-LTT FIIQDRFMSP KQGDNILPTE
Q--LCKKLNT LQEREQFFQ- -LGSIRARKT FIIQDKFNVL KQSPDRIPLE
NVYYPCISSN INTTS---IR VMSVIFEMSN IAHGLSLLDK LQKSSTILAT
NNPLVMNLNS AQQSGYFFRT G--CVLLLTT FIIDI-FNPL QHSQDFISME
TETARGLITD LGSMK---PS DISTFLSFKS -SLQSSILDL FQRSSRALSP
TEMAKGSAKD LDSTRPIRPC DTSALSSLSN V---LSILDL FQRSSKVYVR
HEVQQSIVDE FDSALCFFRT GNTFILGRFS PPQGQDLLSI EDRSTGICGA

SQAVHIEDVA LESVRYKESI IQGLLDTTE
NQALHLEDVV LESHRYKQAV IDHILDRSA
NQAIHLEDVV IESQRYKDSI IARVLDQSE
NQAVHLEDVV LESRRYKELI ISRVLDQSE
NQTVHLEDIV LESKRYKMSV VERLQDKSE
LAGNL--NVI STHINFTTNY MTNFTKSQE
DGVTALQGAL TRREGTRQKS WTSMTGCLE
DNETCIQGAL TMGKGMRQKL WTIIIASLE
LAGSL--NVI STHVGFTTNY MTDFDKSQK
DNESCIKKAC TKNGPLLQPV WNYLYSRIC
LAGSL--NVI STHVGFTTNY MTDFDKSQK
LAGSL--NVI STHIGFTTNN MTDFAKSQK
DNESC--GAL TMGEGMHQKL WTFTTASLE
DNESCIKGAL TMGEGMHQKL WTFTTASLE
LAGSL--NVL STHVGFTTNY MTDFAKSQK
LAGSV--NVI STHIGFTTNY MTDFAKSQK
LAGSL--NVI STHVGLTTNY MTDFAKSQK
PTGSL--NIT LTHVMFITDY MADFAKLQY
HMRTCVHGFA --GEGMRQKL W--------
---------- ---VMFTTDY MADFAKLQR
L------NVI STHIGFTTNY MTDFARSQK
-------NPP TMGEGMRQKL WTFTTASLE
LAGSL--NVI SSHIGFTTNN MTDFAKSQK
LAGSL--NVI STHVGFTTNY MTDFAKSQK
LTGSL--NIT LTRVMFITDY MADFAKLQC
PPHTCIHCTG LAGERMEQEF WAIVTSCLE
DIRTCIHGAS CPGEGVRQNL W--------
SRNYR----- ---KGYEPEV VSTITGCIK
AGSL---NVI LTHVAFTTDY MPNYSKSRD
-------KVI ILSDTVPEVI LTVMSLSVY
LTGLR--YLL TSCLQLILLI LVSQKTTL-
EGETCI-GAL TMGEGGSQKL WTIITGCIE
EGETCI-GAL TMGEGGSQKL WTIITGCIE
DQTPIH-GAK PAGERMIKKL W--------
RQKLCFGDLV SESKQYKLDV INRLLGKVD
IRSICILGTN SSTERSKNM- ---------
YQAKPMSERI MT-QPARKGS WSLPTPSQS
HSRVS--SLQ AKDQGMPTDT FS-------
DFNSEEMYLA QNSWLLRTML EISLSPRNS

3) The data set of EBLLs including non-mammalian sequences.

```
   17    263
Asm.L1     QFFNWQHKIL EQSQIYVADP YCPPSGLDKR EPTFQQKGWT M-----FSIA
Aea.L1     DFSSWNHNFR RETVDETA-- ---GVVLDSW MLALNQATWT F-----MFLA
Pat.L1     DFIKWNLQLR YETLRGVAPP LCATVGKKQK KPHLAQKRWT L-----DNLM
Epf.L1     NEPSALALCS DPTCLNIIPL RRPEIVLKTW EPKFQKSSTV IGISQTMSMR
Myb.L2.1   LYPSALALFA DPTCLNIVPL RRPEVGLKAI EPKFQKSSTV IGISQSMSMR
Myb.L2.4   LYPSAHALCA DPICPNIVLP RRPEIVLKTW EPKFQKSSTV IGISQNMSMK
Myd.L1     LYTSAPALCA DPTCPNIVLL RRPEIVLKTW GPKFQKSSTV IGISQNMSMK
Myl.L1     LYPSALAPCA DPTCLNIVPL RRPEVVLNTW EPKFQKSSTV IGISQTMSMK
Myl.L2.2   LYPSAHALCA DPTFPNIVLP RRPEIVLKMW EPKFQKPSTV IGISQNMSMK
Mod.L1     LEPSPLALCA DPTCLNIQPL RRPEVILKEW DPPFQKSSTV ITISQSMMMR
Ran.L1     IDSSELTLCS DPTL----PL RRPKVVLRVV TPPFQKSSTV IGISQSMPMK
Ran.L2     IDSSELTLCS DPTL----PL RRPKVVLRVV TPPFQKSSTV IGISQSMPMK
BDV        PYPDWLSLVT DPTSLNIAQV YRPERQIRRW MPLFQKSSTV VSASQAVHIE
ABVcoc     AYPDWLSLIT DPTSLNIQQL LRPERQIRKW MPLFQKSSTV VSANQALHLE
ABVduc     AYPDWLSLVT DPTSLNIAQV FRPERQIRKW MPLFQKSSTV VAANQAIHLE
ABVgoo     AYPDWLSLVT DPTSLNIAQV LRPERQIRKW MPLFQKSSTV IAANQAVHLE
RBV        SSTDWLALVT DPTSLNISQL LRPERQLRRW IPLFQKSSTV ISANQTVHLE

           ALLLASTRVG ARISAVVQGD NQCHPDSTWT SR-KRDALTL ARNFHALAEI
           GLKESLEKIG YRYHITVKGD DVRISLLVPS A--ELKTMGF SALRKQLMEE
           ILLFSSFLAQ LQMTITGSGD NNQLPDKQYM EKYENEVIEQ IEEFMNILEK
           SIAQESRTFK DRLISSILAP NTKLRQDTWG VKLEGVSMPF IAEQFHLQET
           SITEGSKRFK DQLIRSILAP NTKLRIATWG CKLHGVTMPF IAEQFALRET
           SITEESKRFR ---------- NTKLRTTTWG AEIHGVTMPF VAEQFIMQET
           SITEESKRFR DQLVRSILAL NTKLRTTT-G TELHGVTMPF VAEQFIMQES
           SITEESKRFR DQLVRSILAP NTKLRTATWG AELHGVTMPF VAEQFIMQEI
           SITEESKRFR DHLVRSILAL NTKLRTTTWG AEIHRVTMPF VAEQFIMQET
           SLTLASRNFL FQVINSIMRP NLRLGHNTGG RKLEGVIKPF IAEQFKLRES
           SIIEESHYFN DKVMASILEI NNRLRRDMLG QTLYIVTISF TDEHFSIHIQ
           SIIEESHYFN DKVMASILEI NNRLRRDMLG QTLYIVTISF TDEHFSIHIQ
           DVALESVRYK ESIIQGLLDT TEQLRRLTWG RDLVGVTMPF VAEQFHPHSS
           DVVLESHRYK QAVIDHILDR SAGLRRLTWG RDLVGVTMPF VAEQFNPVES
           DVVIESQRYK DSIIARVLDQ SERLRRLTWG RELVGVTMPF VAEQFVPCNS
           DVVLESRRYK ELIISRVLDQ SEQLRRLTWG RELVGVSMPF VAEQFTPCDS
           DIVLESKRYK MSVVERLQDK SERLRALTWG RELVGVTMPF VSEQFRPVKS

           KESETIISSS FFIYGKRILR EGAMLPQCLK AASRILLHAD TLLDDTRSAL
           NPNESFVSLS IVCTSKQYIV RDTWLPSAAK KILKCGAVTN VVFPTLSDHV
           KREETGISQN VFIYGKEILV FGAFLPGALK KIGRVYFDVN EIYPTCESKL
           RPDTKLFPRQ WTKRGSRPLY IGSRTHIKVN RGAITGLPEG KIGKMVEDLI
           RPDNHLFPRE WMVRGKHPLY IGSRTFVTIN RGAITGLPEG PIGKMAEDLI
           RPDE-LFPRL SMIRGKRPLY IGSRTFAKIN REAITGLPEG RVGKMAQDLI
           RPDEELFPRL WMIWGKRPLY IGSRTFVKIN RGAITGLPEG RIGKMAEDLI
           RPDEELFPRL WMIRG-RPLY IGVKICKKIN RGAITGLPEG LIGKMA-DLI
           RPDE-LFPRL SMIRGKRPLY IGSRTFANIN REAITGLPEG QVGKMAQDLI
           RPEDEILPRD -THRGSRPLY IGSRTFVKIN RGCL-KVNLG K---MA-DIV
           RPDDLIQPWT WTVRGRRPLN IGSQIFVKIY PGADIRLLEG E-----RELK
           RPDDLIQPWT WTVRGRRPLN IGSQIFVKIY PGADIRLLEG E-----RELK
           CPQETLRSHH LTTRGDQPLY LGSNTAVKVQ RGEITGLTKS RAANLVRDTL
           VPQEPLRERH LYIRGSQPLY LGSNTAIKVQ KGELTGLSKS RAAGLVRDTL
           CPQEPLRMRH LSTRGDQPLY LGSNTAIKVQ KGDITGLNKS RAASLVRDTL
           CPQEALRMRH LSTKGDQPLY LGSNTAIKVQ KGDITGLNKT RAANLVRDTL
           CPEEPLTPAH LDKKGKQPLY LGSNTSVKVQ KGEITGLNKS RAAGLVRDTL

           STVATSCTKV LNDPRLCFLL TLFHSCRQLA HSLVIADLKR LCGLLPLDWA
           ATIYSVAHSA CAVALPCFVT ACYMAARLLV REMHLKEHLL VLNLWPQVLG
           VTIHTSSQAA AQNPLISYAI ALIASYYSLL QDLKLLKFVR FCCLYPRSLG
           LTCNWIKLRG SVGGNMANLL LQLLKEKGVT SPTLISTHIN FTTNYMTNFT
           STCDWIKIKG AGGQHLARLL SILLSEKGVH TPDLISTHVG FTTNYMTDFD
           STCDWIKMKG AFGQHLATLL STLLSEKGVH PQELISTHIG FTTNNMTDFA
           PTCDWIKIKS AFGQHLAMLF STLLSEKGVH APEFISTHIG FTTNYMTDFA
           STCDWIKIKG AVGQHLATLL STLVSEKGVH APELISTHIG FTTNYMTDFA
           STCDWIKMKG AFGQHLATL- STLLSEKGVH PQELISSHIG FTTNNMTDFA
```

PACEWIKLQG ETGENLAELF TTLLKEKSIT PPSLILTHVA FTTDYMPNYS
ILLHYVTGSS LEGPNLVELL HKFLREKGVC LPSLMSAKVR VTTDYMLNFA
ILLHYVTGSS LEGPNLVELL HKFLREKGVC LPSLMSAKVR VTTDYMLNFA
VLHQWYKVRK VTDPHLNTLM ARFLLEKGYT SDARLSTWLR FSSDYLHSFS
ILYQWYKVRK VIDPNLNKLM DRFLQEKGYA SDARISTWLK FSSDYMSTYS
VLYQWYKVRK VIDPNLSKLM DCFLREKGYT SEIRLSTWLR FTSDYLQTYS
VLYQWYKVRK VVDPNLSKLM DCFLKEKGYV SEVRISTWLR FTSDYLQTYS
VLCEWYKVRK INDPNLSLLL KRFLTEKGYN QTQSISTWLR FTSDYLTTYS

NRFLDQVSDL SWAQWGADPY IPSTMTLTKI VEDLTQRGVL SNARNPVLKD
---GPGVLPL QTFFVRGEND LSCSISLMRM LDLVILESIV RSACRSILNQ
----GFVSTL LSYLYRGHPD VTEGLSFLKI LG--HELKLY KNAMQYIIQD
KSQEDYTIHF QAAFLHGLNL LASKLHYGTL EPITYYLTTS CKTCTSLIEE
KSQKDYTIHV QDAFLHELNL LSSQLHLGIL ANKTYYLTLA CTGYTREIQE
KSQKDYTIHF QAAFSHGLSL LSSQLHFGAL ANKTYYLTIN CPGCTNEIKD
KSQKDYTIHL QAAFSHGLSL LSSQLHFGAL ANKT-YLTIN CLGCTNEIKE
RSQKDYTIHF QAAFLHGLNL LSSQLHFGAL ANKTYYLTIN CPGCTKEIKN
KSQKDYTIHF QAAFSHGLSL LSSQLHFGAL ANKTYYLTIN CLGCTSEIKK
KSRDDYTLHF QAAFIHGINM LAYKAHYGTI KKETYYLAIN CTKCTRKIEK
NSQKDYLIHF PVAFKHVFNL LTSQV----- -----YLTGF CSKCVNSLQE
NSQKDYLIHF PVAFKHVFNL LTSQV----- -----YLTGF CSKCVNSLQE
KSSDDYTIHF QHVFTYGCLY ADSVIRSGGV ISTPYLLSAS CKTCFEKIDS
QSSEDYTIHF QHVFTYGCLY ADVMVRSGKI IREPYLLTAS CKTCFEKIES
HSSDDYTIHF QHVFTFGCLY ADSVIRSGGI ISKPYILSAS CPTCFEKIES
RSSDDYTIHF QHVFTFGCLY ADSIIRSGGV VSSPYILSAS CQTCFEKIES
KSVDDYTIHF QHVFTYGCLY ADSVLRSSGH IQEPFLLEAA CSSCFERITS

LFHSDAKAED ERI
PLENPPNHKQ LLL
PIFKSTQDLT MLL
GGFELTRPAA YKG
DFFSLSRPPM YKG
-SFDPAVDAG FKF
DPFSLSTSPI YKG
-SFSLSKSPI YKG
DSFDPAVDAG FKF
-FFSLLKRPQ YQG
WSFYLDREPC TEM
WSFYLDREPC TEM
EEFVLACEPQ YRG
EEFVLAVEPQ YQG
EEFLLACEPQ YRG
EEFLLACEPR YKG
EEFVLASSPM YQG

4) The EBLNs used for molecular dating.

12 69

Epf.N7 LKLFASFAEM TPYMTMKALI AESLSEIMLL PLIALEATKL SDAEKELQEM
Epf.N8 LKLFSTFSEM TPYVVMKALI NDGIGHVMTL RSVADDAAKL VTVERELMEK
Eih.N1 VKMVAATPRQ WPTWS-GSIL DQCLEETFVV PRVTAEIQRG CESL------
Epf.N3 LKLFSAYAEM TPYLTIRAFI DEGLTRAMFL PKVGGETRKF LDAEEALQRQ
Epf.N6 LRLIATFAEM TPYLTIKAFF NDGYTKAMTL PGVGDKMGAF VDTEKLLKER
Epf.N4 LSLVASFAEM TPYLTIKSFF GDGYTKGITL PGVGDEIGAF VDAEKMLKER
Epf.N9 LRLAASFAEM TPYLTIKSFF GDGYAKGMTL PGLGDKIGAF VEAEKSLKEC
Epf.N10 IRLISSFAGM TTYLTIKEFI NEGLSKASLL PGVDSEIKAF V-----LKQR
Mel.N12 VKVCLNMPRQ QQRSAVRGHQ DQCRDGTIEV PQAAAVIQPF LAPVEEVKEV
Myd.N5 IKLVATYAEM TPYMTMKSFI NEGLSYGAML PGVATEIKAF LQVERELKEE
Myd.N1 VKLVATDAEM TPYMTMKSFI NEGLSYGAML PGVATEIRAF LQAERELKEE
Ptp.N3 VHMVVREAQM TALIAINTFL KEFNSVLLLI PGPMDEIPKF HEAWRHLEQQ

SSAAKFPYL- VLTLKHHDL
HTKEKFPYLK ILRLEGHER
RSL---CLCE VPHSPQFQT
HGEELFPYIK VLRLKGHET
HGVAIFPYCK VLHLAGHEA

HGETMFPNCK VLHLAGHEA
H-ETMFPYCK VLRLTGHES
HGYELFPYLK ALKLQGHEA
HK-QMLSGTT SQELNFHPR
HS---FTDMS CWHLFGFLT
HGEELFPYLK ALKLRGHEL
IGAKLFPYVK AIHHPEAAK

Data S2. Alignment of paired SINEs between two bats. Alignments of SINE-A sequences and SINE-B sequences are shown. The A1 and A2 represent a duplication of SINE occurred in Ptp (GenBank number AWGZ01242856.1; 5858 bp) and B1 and B2 represent a duplication of SINE occurred in Myd (ALWT01098736.1; 10,425 bp). Asterisk (*) denotes same nucleotide in alignment.

```
               *********  ** ******* * *****           * ***  ***
Ptp SINE-A1 CCCTGGCCAA GTAGCTCAGT TGGTTAG--- ---------A TATGCCAATG
Ptp SINE-A2 CCCTGGCCAA GTTGCTCAGT T-GTTAGACT GTGGTCTCAA TATACCAAGT
Myd SINE-A  CCCTGGCCAG GTGGCTCAGT TGGTTAGACC GTTGTCCCAA TATA-CAAGG

            ***   ***   *********  ***  ** * *  ******  ***** ** *
            TTGTGGGTTT GATCCCCAGT GAGGGTACGT ACAAGAATCA ACCAAGAACA
            TTGCCAGTTC GATCCCCAGT CAGGGCACTT ACAAGAATCA ACCAATAA-A
            TTGCGGGTTC AATCCCCAGT CAGGTCACAT ATGAGAATCT ACCAAGAACA

             **
            AGTGAT
            TGTATC
            AGTGGC

             *** * ***   ** ***** *    ** ** *     ***     *
Myd SINE-B1 GCCTGGCCAG CTTGGCTCAG TGG-TTG-AG CGTCAACCTA TGAACCAGGA
Myd SINE-B2 CCCTAGTCAG TGTGGCTCAG TTGATTGAAG CACCGTCCTG TGCATGGAAA
Ptp SINE-B  CCCTGGCCAG TGTGTCTCAG TGGACTG-AG CTCTGGCCTG CAAACCAAAA

            ***  *  *  * **** *    *  *****  ** * ** ** **  *
            GGTCACG-GT TTGATTACTG GTCAGGGCAC ATGCCCAGGT TGCGGACTCG
            GGTTGCAGGG TCGATTCCTG ATTGGGGCAC ATACGCAGGT TGCAGGTTCA
            GGTCGCATGT TCGATTCCCA GTCAGGGCAT ATGCCCAAGT TGCGGGCCAG

             ****  **         *      ** **  *  ** ****     ***  *
            ATCCCCAGT- GCAGGATGTG GAGGAGGCAG CTGATCAATG ATTCTCTGTC
            GTCCCTTGTC TGGATGCATA TGGGAGGCAA CCGATTAATG TTTCTCTCTC
            GTCCCTAGT- TGGAGGTGTG TGATAGTCAA CCTATCAATG --TATCTCAC

            * *** * ** ***** ** * ****   **   *    ***   **** *
            ATCATTGATG TTTCTATCTC TCTCTCCCTC TCCCTTCCT- -CTCTGAAAT
            A-CATCGATG TTTCTGTCTC TCTCCCTCTT CCCTCCCCTG TCTCTAAAGT
            A-CATTGCTG TTTCTCTCCC TCTCTTCCTC CCTTCCCCTC ACTCTAAGAG

             *  *****  * *  *          * *   ** *
            CAATAAAAAT ATAT--TTAA AAACAAAATA AAACA
            TGATAAAAAT GTATCCTCTG GTGAGTATTA AAAAA
            TAAATAAAAT ATTT--TTAA AAAAGATTT TAAAA
```

Data S3. Alignment of paired bat sequences containing EBLLs. Query, Ptp (GenBank number AWGZ01242856.1; 5858 bp); Sbjct (subject), Myd (ALWT01098736.1; 10,425 bp). The numbers are consistent with the contig numbers. Vertical bar (|) denotes same nucleotide in alignment.

```
Query  621  ACCCACAAAAGACTAAAGAAGGGAGGCCAGCAAAATTCAGTGGTCTTCCTTCCCCCTCA-  679
            ||||||||||| ||| ||||||||||||||||||||| | ||  || |  | || |||
Sbjct  3    ACCCACAAAAGTCTATAGAAGGGAGGCCAGCAAAATTAAATGCCCTACACACACCTTCAA  62

Query  680  CCACAAGAGGAAGTT--GGCGTGGTAGAACAAAATGAGACTGGTGATACTAATTGATCAA  737
            || |||||||||   || ||| ||||||||||||||| |||| ||||| ||||| |||
Sbjct  63   CCGTAAGAGGAAGACAAGGATTGG-AGAACAAAATGAGATTGGTAATACTGATTGACCAA  121

Query  738  GTGACAT-TTTTTATACATCCTAGTTTTAGCATATCAATGGTAGTCCTTCTAAATGACCT  796
            || | || ||||| |||||||||||||||| || |||||| || || | |||||||||||
Sbjct  122  GTTATATATTTTTCTACATCCTAGTTTTAGTATTTCAATGATAATCGTGCTAAATGACCT  181

Query  797  TTCTGATAGTCCCAGGAAAGCGAACAAGACATTCTA  832
            |||||||||||||| ||||| || ||||||||| ||
Sbjct  182  TTCTGATAGTCCCAGAAAAGCTAAGAAGACATTATA  217


Query  826  CATTCTATCCTTCCAAGGAAATTGTTTTGTAGGTAACCCAATGTATAGTCTTGCTGCCCT  885
            |||| || ||||||||||||||| | |||||| |||||||||||||||||| || ||||
Sbjct  432  CATTATACCCTTCCAAGGAAATGGCCTTGTAGATAACCCAATGTATAGTCTTTCTACCCT  491

Query  886  ACTCTCAGGTCTGAGAGCCTGCCATTTTCTAACCTCTGCCATGGAGCTATCCATCTGTCT  945
            ||||||||||||| |||||           |||||||||| ||| ||||||| ||
Sbjct  492  ACTCTCAGGTCTAAGAGC----------------TCTGCCATGGGGCTGTCCATCTCCCT  535

Query  946  TTGGGAAGTTGTATTTGGTCTCAATCTAGGAACTG----------CTTTCAGCAGTTCAT  995
            ||||||||||| || ||||| ||| | ||||||||||       ||| |||||| ||||
Sbjct  536  TTGGGAAGTTCTAGTTGGTTTCATTTTAGGAACTGTCTCTTGGTGCTTCCAGCAGGTCAT  595

Query  996  TCTCTCAGCCTGACTTA----GTCAAGGAAACCCAAATGGGCCCTGGCCAAGTAGCTCAG  1051
            |||||||| |||||||  |||||| |||| ||||||||||||||||| || ||||||
Sbjct  596  TCTCTCAGTGTGACTTATCCAGTCAAGAAAACTCAAATGGGCCCTGGCCAGGTGGCTCAG  655

Query  1052 TTGGTTAGA----TATGCCAAT-------GTTGTGGGTTTGATCCCCAGTGAGGGTACGT  1100
            |||||||||  | | ||||||    |||| ||||| ||||||||| ||| || |
Sbjct  656  TTGGTTAGACCGTTGTCCCAATATACAAGGTTGCGGGTTCAATCCCCAGTCAGGTCACAT  715

Query  1101 ACAAGAATCAACCAAGAACAAGTGATACAGGTTACAAAAAAGACATTCTAG--CAGTACA  1158
            | |||||| ||||||||||||||| |||||| ||| ||||| ||||||  ||| ||
Sbjct  716  ATGAGAATCTACCAAGAACAAGTGGCACAGGTCACAGAAAAGGTATTCTAATTCAG--CA  773

Query  1159 ATTATTCCTCAGTTACTCAAGGTTG-TTTATTTGGTTGTTAGCCTCTGCATATAACTTTT  1217
            |||||| |||| ||||||||| || ||||||||||||| || |||||| |||||||||||
Sbjct  774  ATTATTTCTCAATTACTCAAGATTAATTTATTTGGTTGTGAGACTCTGCCTATAACTTTT  833

Query  1218 TC-CTCTTTTTCACTTCTTACCCTCCATCTGCTATTCTTAGTGTAAAGCATTAATATTGG  1276
            || ||||||| || | |||||       || ||||| | | ||||||||| ||||||
Sbjct  834  TCTCTCTTTGTCCTTCCTTAC-----------TACTCTTAATATGAAGCATTAGTATTGG  882

Query  1277 GACACCTGATCTGTGAAGACAGTGGCCACAGAGGAGTTATGAATTCCAACCAAGTAGATC  1336
            |||||| | || |||||||| ||| ||||||||||||| | || |||| || |||||
Sbjct  883  GACACCCAACCTATGAAGACAATGGAAACAGAGGAGTTATCAGTTACAACTGAGCAGATC  942

Query  1337 AATAGAACTGATCAAACTCTTA  1358
            |||||||||||||||||||||
Sbjct  943  AATAGAACTGATCAAACTCTTA  964
```

```
Query  1353  CTCTTAAGTCCCCTTTACGCTCTACCAAAGTAGAGTTTTGCCTCAAATTTCT--AGTTAA  1410
             |||| ||||| ||| ||| |||||||||||||||| |||||||||||| |||  | ||||
Sbjct  1198  CTCTAAAGTCTCCTATACTCTCTACCAAAGTAGAGCTTTGCCTCAAATCTCTCTAATTAA  1257

Query  1411  ATTTGACCTGCATAAACATGTTGTACAATATGTGACCAACATTAACTCTGATTCCATCAG  1470
             | ||||||  ||||||||||||  | ||| | |||||||||||||||||||||||||||
Sbjct  1258  ACTTGACCACCATAAACATGTTCAAAAATCTATGACCAACATTAACTCTGATTCCATCAG  1317

Query  1471  CATTGCACTATCCAATATTTATCCCTTATTTCTGCTCTCAGGCCTTGTTCTCATGGCACA  1530
              ||| |||||| |||||||||| ||||| || ||||||| ||||| ||||| || |
Sbjct  1318  AATTATACTATCGAATATTTATCTCTTATATCCTCTCTCAGACCTTGCTCTCACTGCT-A  1376

Query  1531  CTGTGGCTGCCACAGTGGCAGGCACACGGGTGACAACAGTCAGAGCATGGCAAGGTGATA  1590
             |||  || ||     |||||||| |||  | ||||| |||||| |||||| ||||||
Sbjct  1377  TTGTCA-TGGCA-----GCAGGCACT-GGGCAATAACAGCCAGAGCGTGGCAATGTGATA  1429

Query  1591  TAGACTTAAAATTAATTG-----CAAGCAATCAAAAATTGC--CTCTTGTCTAGACATTG  1643
              || ||||||  | |||     |||||||||||||| |||  |||||||||||| |||
Sbjct  1430  CAGGCTTAAAGGAAGTTGTATCACAAGCAATCAAAAGTTGTATCTCTTGTCTAGATATTA  1489

Query  1644  TGACAATTATTGGGAAGCATATTCTTGTGCAAGAAATTATAAAGAATGTTAGTTTCACCC  1703
             |||||||||||| |||||||||| |||| ||||||||||||||||  | || ||| |||
Sbjct  1490  TGACAATTATTGAGAAGCATATTTGTGTGTAAGAAATTATAAAGAAAATCAGCTTCGCCC  1549

Query  1704  ATACAAAAGACTTCATTGAATTATGGCTTTGGG-ACCCTGAACTA---AGTTTCATCCAG  1759
             |||||||| |||||| || ||| |||||||||| |  ||||||   ||||||||||||
Sbjct  1550  ATACAAAAAACTTCACTGGATTGCGGCTTTGGGGAGTTTGAACTCCCCAGTTTCATCCAG  1609

Query  1760  TCTTTGGTTATGGTAAAATTGTATTATACCAACCAAGAATGTTTAATGACTTATAACCAT  1819
             ||||| ||| || ||||||  ||||||| ||||||||||||||||||||||||| ||||
Sbjct  1610  TCTTTAGTTGTGTTAAAATCTCATTATACAAACCAAGAATGTTTAATGACTTATAGCCAT  1669

Query  1820  TTCCTTGCATTTGCTGATA-TGTCAAATCCCAAATGAACTTCATGATTTCCAGTTTCATC  1878
             ||||||||||||||||||| |||||||| || |||||||| |||||||||||| ||||
Sbjct  1670  TTCCTTGCATTTGCTGATACTGTCAAATTGCAGATGAACTTTGTGATTTCCAGTTCCATC  1729

Query  1879  AGTGACTCTGTCCACAGGACCTTGACATATACTTATCCTACAGTACTAAAAATCTTAGCG  1938
             |||||||| || ||||||||||||| || |||||||||| |||||||||||| || ||
Sbjct  1730  AGTGACTC-GTTCACAGGACCTTGATATCTACTTATCCGCCAGTACTAAAAACCTCAGAA  1788

Query  1939  TGGACTGACAGTGCAGCTATGGTCCTTACAACAGATGACTACTTTGAAGGGATAAAATCA  1998
             |||||||| ||||| || |||||||| ||||||||| ||||||||||||| ||||||||
Sbjct  1789  TGGACTGATAGTGCGACTGTGGTCCTTGCAACAGATGGCTACTTTGAAGGGCTAAAATCA  1848

Query  1999  ATTTTACCTTTCTGTCAGGGATGTGTTACGGAAGTACACAATAGTCCATCACTTCCCTCA  2058
              ||||||||||| ||| |||||||||| ||||||||| |||||| |||||||| ||||
Sbjct  1849  GTTTTACCTTTCTATCAAGGATGTGTTATGGAAGTACATAATAGTGCATCACTTTCCTCG  1908

Query  2059  AGCTTTAAAGAAGTAACAAGCAGTGCCATAGACAAATTATCCCAGCAGCTTGAGCAGACC  2118
             |||||||||||||||||||||||||||| |||||||||||| |||  | |||||||
Sbjct  1909  AGCTTTAAAGAAGTAACAAGCAGTGCCATAAGCAAATTATCCCAACAGTGTAAGCAGACT  1968

Query  2119  ATAGAGA--CTCTCAGCAACTATACCA-CTGTGGCTCTGGAAATATGCTCAGTAAATAAA  2175
             |||||||  |||||| ||||||||||| |||||||| |||||||| || ||| |||||||
Sbjct  1969  ATAGAGACTCTCTCAACAACTATACCAGCTGTGGCTGTGGAAATAAGCCCAGGAAATAAA  2028

Query  2176  GTAAATAAACTTCTTTCCTGCAACTGGCTT-------------------------AGTTC  2210
             |    || || |||| |||| | || |||                          |||||
Sbjct  2029  G----TACACGTCTTCCCTGAGATTGACTTGACTTAGAATCTGGTGTTACTGAGCAGTTC  2084

Query  2211  AGTAAGATGAGGGCTGAGAAAGGAGACTCTCAGTCACTCCTTTCATATGGATCTTGACTG  2270
             |  |||||||| ||||||| ||||||    || ||||||||||||||| |||||  |||
Sbjct  2085  ATCAAGATGAGAGCTGAGATAGGAGA----CATTCACTCCTTTCATATTGATCTCAGCTG  2140
```

Query 2271 CACAATGTGTTTGTTGCTGAATATATTAGGGGGCATCTGTATAAGCATGG-GATGTGA-- 2327
|||| ||||||||||||||||||||||| |||||| || |||||||||| || | |
Sbjct 2141 CACATTGTGTTTGTTGCTGAATATATTA-GGGGCACCTACATAAGCATGGAGAACTCATG 2199

Query 2328 CCTTC-GTGAAAGATCTTGTTACCAGGACTTGCCCACTAATGCTCTATTGCAGCTTAGTC 2386
|||| ||| ||||||| | |||| |||||||| ||| ||||||||||| ||| | |
Sbjct 2200 GCTTCTGTGCAAGATCTCATCACCAAGACTTGCCGGCTACTGCTCTATTGCGACTTGGCC 2259

Query 2387 AAGGCAGTATAAATATGATGACTTCCAAGATGTTTCCTTAGACACATCATCTACCTCCTT 2446
|| | ||| || || |||||||||||| |||||||||| |||||| || ||||
Sbjct 2260 AATGGAGTTCAAGTAGTGTGACTTCCAAGATATTTCCTTAGATGCATCATATATTCCCTT 2319

Query 2447 GGATCTAAATCCAGATATCAATGATCTGTTATCTGACACATTGATAATAGAGTCAAGGAA 2506
||||||||||||||||||| |||| |||| || | |||| |||||||||||
Sbjct 2320 AGATCTAAATCCAGATATCAACAATCTAGTATCCCCCAAGTCCATAACAGAGTCAAGGAC 2379

Query 2507 ACACTGGAAGTATGAATTTAACATTGTAGCATACAGAGAAAAGAACAAGCAACCCATACA 2566
|||||||| | ||| ||||||||||| ||||||||||||||| |||||||||||| |||
Sbjct 2380 ACACTGGACATTTGACTTTAACATTGTGGCATACAGAGAAAAGCACAAGCAACCCAAACA 2439

Query 2567 ACATCCAGC-AAACACAGCCTCAAACGTTTTGTCAAAGCTTTAGTTGATGGTCATTTGGT 2625
||||| ||| ||||| |||||||| || ||||||||||||| |||||||||||||||
Sbjct 2440 ACATCTAGCAAAACATGGCCTCAAATGTCTTGTCAAAGCTTTGGTTGATGGTCATTTGGA 2499

Query 2626 TAACGTTCCTGCAATGCTGGCTCCATTTTGCGTAGGGAAATTGAAGTCCGGAATGTGTTT 2685
||| |||||||||||| ||| |||| |||||| |||| ||||| | || | |||| | |
Sbjct 2500 TAATGTTCCTGCAATGTTGGTTCCA-TTTGCGCAGGGTAATTGGACTCTGAAATGCATAT 2558

Query 2686 CACAGTTCTGGTTCCTAAGGAGAAAGAACTCAAGAGAAAGGCAAGATTCTTTAGTGTTCA 2745
| ||| |||| ||||||||||||||||||||||||||||| | || ||||||||||||
Sbjct 2559 CGCAG-TCTGTTTCCTAAGGAGAAAGAACTCAAGAGAAAAGGGAAATACTTTAGTGTTCA 2617

Query 2746 GTCA 2749
|||
Sbjct 2618 ATCA 2621

Query 2979 aataatTACATCACCAAGTGTATCAAGTTGTAGCAGAGATTTATATCAAAGAATGCGTAA 3038
||| |||||| ||| |||| |||| || |||| |||||| |||| ||| || | | |
Sbjct 2617 AATCATTACACCACTAAGTATATCGAG--GTAGTAGAGATGAATATAAAAAAACACATCA 2674

Query 3039 TGCCACTAATCCA-----------AATG---------CAGTTAAATCTTGTTCTTGCAAA 3078
||||||||||| |||| ||||||||| || |||||| |||
Sbjct 2675 TGCCACTAATCATTGTTTAATGACAATGACTTCTACCCAGTTAAATAATGCTCTTGAAAA 2734

Query 3079 ACTATCTCATATTGCCACAATACTTAATAAGTTTGTTATCAATCTGGATTCCTCATCATG 3138
|||| ||||| |||||| || | |||||||||||| || || |||| |||||||||
Sbjct 2735 ACTACCTCATGTTGCCAGAAGGCAGGATAAGTTTGTTATTAAACTAGATTACTCATCATG 2794

Query 3139 GTGTAATTACTTTA-ACCAGAGATCCAGTATGCATTGTGTGCAGAATCGGATGTCTTTCT 3197
||||||||||||| ||||||| || ||||||||||| |||||||| ||||| ||||
Sbjct 2795 GTGTAATTACTTTAGACCAGAGGTCTAGTATGCATTGGGTGCAGAAATGGATG---TTCT 2851

Query 3198 TGGATGTGACAAATTCTTTAGGACAGGGAACATTATGCTAAGATATACCA 3247
|||||||| ||||||||| |||||||||| ||| ||| || |||||||
Sbjct 2852 TGGATGTGGTGAATTCTTTAAGACAGGGAACCTTACGCTGAGGTATACCA 2901

Query 3250 TTTACTACACAAGATAAGTTCAACCTTCCCTGGCAGTTACCTGATGGATTGCCTGTGGAG 3309
|||| || |||||| |||||||||| |||||| |||||||||||| | ||||| |||||
Sbjct 3309 TTTATTATACAAGACAAGTTCAACCCTCCCTGTCAGTTACCTGATAGGTTGCCCATGGAG 3368

Query 3310 GACATAAGAACCTGCATACACGGTGCCAGCTGTCCTGGAGAAGGGGTGAGACAAAATTTG 3369
|||| |||||||| |||| || || | ||||||||||| |||| ||||| ||
Sbjct 3369 CACATGAGAACCTGTGTACATGG------CTTTGCTGGAGAAGGGATGAGGCAAAAGTTA 3422

```
Query  3370  TGG----------CCTCTTACTCAAAATTTTCAGCTTTTGAAGAAGTTGAGATCAAGGG  3419
             |||          |||||| |||| || | |  |||| ||||||| |||| | |||||||
Sbjct  3423  TGGACTACAATGGCCTCTTGCTCAGAACTAT--AGCTCTTGAAGATGTTGGGGTCAAGGG  3480

Query  3420  TGTTATTCTTGACCAGGCTGACAACCGAACAATAGTTGTTACCTGT  3465
             ||||||||||||||||| ||||||| |||||| |||||||||||||
Sbjct  3481  TGTTATTCTTGACCAGGGTGACAACTGAACAACAGTTGTTACCTGT  3526


Query  4682  TAATCACTCGTTTCTTACAGCTTGTCTTTCTTCCTTACCCATGATTCCTATACCAACATT  4741
             || ||||| ||||||| |||||||||| |||| | ||||||||||||||| ||| |||
Sbjct  4092  TAGTCACT-GTTTCTTGAAGCTTGTCTTCCTTCTTAACCCATGATTCCTATCCCA-CATG  4149

Query  4742  TGCTTCTTGTGTCATGCTTTCATCATTACCACCATCACCAATAGCCAATGTTACAGGTTT  4801
             ||       |||||||||| ||||||| |||||||| | ||||||||||| ||||||
Sbjct  4150  TG-------TGTCATGCTTACATCATTAGCACCATCATTACCAGCCAATGTTATAGGTTT  4202

Query  4802  CTCAGGTGAGGTT-------------ATACCTTGGATTTCTGCAGTGGTACCATACGACA  4848
             |||||||||| ||             | || ||| ||||||||||||| ||||| ||||
Sbjct  4203  CTCAGGTGAGATTTTTATCAGCTTTATTTCCCTGGCTTTCTGCAGTGGTGCCATATGACA  4262

Query  4849  GTCCAATGCAGCTGAACAATTTCTGCAAACACCCATAATAGTTTTTCATGCTCTCAGAAT  4908
              | |||| ||||| |||| |||||||||| | ||   ||||| ||| ||||| | || |
Sbjct  4263  ATGCAATACAGCTAAACAGTTTCTGCAAATATCC---ATAGTATTTTATGCTACCTGAGT  4319

Query  4909  GCAAGAGATACACTGGTCCCATCTACCACCAGCCCATGCTAATTCATGCGATCCTCTAA-  4967
             |||||||||   |||||||||| |||||||| ||||||| | |||   | |||||||
Sbjct  4320  GCAAGAGATCGGTTGGTCCCATCCACCACCAGTCCATGCTGAATCACAGGCCCCTCTAAT  4379

Query  4968  ---TTCTAGCAGGCTACCTCAACTGTT-CGCACATTCACTG----ATTCCTTGTTTATAA  5019
                |||| ||| ||| ||||| ||||| ||||| ||||||    |||||| ||||||||
Sbjct  4380  TCCTTCTGGCAAGCTGCCTCAGCTGTTGAGCACACTCACTGTCTAATTCCTGGTTTATAA  4439

Query  5020  GTTCAAGGATATTCATGGTTCTGTGGAAATGTCCATATGATAAACTCGCACTAACAGCTG  5079
             |||||| |||||||||||| |||| ||||| ||| ||||||||||| ||||| ||||||
Sbjct  4440  GTTCAACCATATTCATGGTTTTGTGAAAATGCCCACATGATAAACTCACACTAGCAGCTG  4499

Query  5080  TTTCATGTTTATCTCCGTTAAGTACCCCATATTTGATACCAACCATTCTCAGTGCTTCA  5139
             || ||||||||||||| |||| |||| ||| |||||||||| || |||||| |||||||
Sbjct  4500  TTCCATGTTTATCTCCAGTAAGAACCCTATACTTTGATACCAGCCGTTCTCAATGCTTCA  4559

Query  5140  ATAGTT--TTGAACTTTATCTTGTAGTCT---CTCTCCTGCTATAGCTCCAAGTAATGTC  5194
              ||||| ||||||| |||||||||||||   ||| ||||  ||||||| |||| ||
Sbjct  4560  CTAGTTTCTTGAACTGTATCTTGTAGTCTGCCTTCTATTGCTGGGGCTCCAAATAATATC  4619

Query  5195  AGGTCTTCCCCCATGAGCTAGCTGGAAGACATTTGCCAATTTTTCTTCCTGTTGCTGCAA  5254
              | ||||| | | ||   || ||||||||||||||||| || |||||| || || |||
Sbjct  4620  ATGTCTTTCTCTAT----TAACTGGAAGACATTTGCCATGTTCTCTTCCCATTCCTACAA  4675

Query  5255  GACAGTTCTGGCTTCAAATAGGCATCTATCTATTACTTCATACTCTTCAGTTGTCAACTG  5314
             | |||| |||||||||||||| |||||||||||||| |||||||||| || ||||||||
Sbjct  4676  GGCAGTCCTGGCTTCAAATAGACATCTATCTATTACCTCATACTCTTTAGATGTCAACTA  4735

Query  5315  TCTGTAGGCCATGTGCAGAGGTCTTAAGCCTTTCAAAGCAAATTCATCTACATGAATTGT  5374
             | |  | |||| |||  ||||| |||||||||||||||||| ||||||||||| |
Sbjct  4736  T-AGCCAGTCATGCACAGGTTTCTTAGCCCTTTCAAAGCAAATTCATTTACATGAATTCT  4794

Query  5375  GGGGT----TTTTTATTTGTCCACCTATACA--TAGGGAGAATTGATGATTCAG  5422
             |  |    ||||||||||||| |||||||| ||||||||||| |||||||||
Sbjct  4795  GATTTTTTATTTTTATTTGTCCTCCTATACATTTAGGGAGAATTCATGATTCAG  4848
```

© 2015 by the authors; licensee MDPI, Basel, Switzerland. This article is an open access article distributed under the terms and conditions of the Creative Commons by Attribution (CC-BY) license (http://creativecommons.org/licenses/by/4.0/).
